# Structural insights into a cooperative switch between one and two FimH bacterial adhesins binding pauci- and high-mannose type *N*-glycan receptors

**DOI:** 10.1016/j.jbc.2023.104627

**Published:** 2023-03-20

**Authors:** Eva-Maria Krammer, Clarisse Bridot, Sonia Serna, Begoña Echeverria, Shubham Semwal, Benoît Roubinet, Kim van Noort, Ruud H.P. Wilbers, Gleb Bourenkov, Jérôme de Ruyck, Ludovic Landemarre, Niels Reichardt, Julie Bouckaert

**Affiliations:** 1 Unité de Glycobiologie Structurale et Fonctionnelle (UGSF), UMR 8576 CNRS and University of Lille, Villeneuve d'Ascq, France; 2 Glycotechnology Group, Basque Research and Technology Alliance (BRTA), CIC biomaGUNE, Donostia, Spain; 3 GLYcoDiag, Orléans, France; 4 Laboratory of Nematology, Plant Science Group, Wageningen University and Research, Wageningen, The Netherlands; 5 European Molecular Biology Laboratory (EMBL), Hamburg Unit c/o DESY, Hamburg, Germany; 6 CIBER-BBN, Donostia, Spain

**Keywords:** FimH, *N*-glycan, multivalency, paucimannose, oligomannose-3, core fucose, oligomannose-6, kinetics, crystal structure, bacterial adhesion, cooperativity

## Abstract

The FimH type-1 fimbrial adhesin allows pathogenic *Escherichia coli* to adhere to glycoproteins in the epithelial linings of human bladder and intestinal tract, by using multiple fimbriae simultaneously. Pauci- and high-mannose type *N*-glycans are natural FimH receptors on those glycoproteins. Oligomannose-3 and oligomannose-5 bind with the highest affinity to FimH by using the same Manα1,3Man branch. Oligomannose-6 is generated from oligomannose-5 in the next step of the biogenesis of high-mannose *N*-glycans, by the transfer of a mannose in α1,2-linkage onto this branch. Using serial crystallography and by measuring the kinetics of binding, we demonstrate that shielding the high-affinity epitope drives the binding of multiple FimH molecules. First, we profiled FimH glycan binding on a microarray containing paucimannosidic *N*-glycans and in a FimH LEctPROFILE assay. To make the transition to oligomannose-6, we measured the kinetics of FimH binding using paucimannosidic *N*-glycans, glycoproteins and all four α-dimannosides conjugated to bovine serum albumin. Equimolar mixed interfaces of the dimannosides present in oligomannose-6 and molecular dynamics simulations suggest a positive cooperativity in the bivalent binding of Manα1,3Manα1 and Manα1,6Manα1 dimannosides. The binding of core α1,6-fucosylated oligomannose-3 in cocrystals of FimH is monovalent but interestingly the GlcNAc1—Fuc moiety retains highly flexibility. In cocrystals with oligomannose-6, two FimH bacterial adhesins bind the Manα1,3Manα1 and Manα1,6Manα1 endings of the second trimannose core (A-4′-B). This cooperative switch towards bivalent binding appears sustainable beyond a molar excess of oligomannose-6. Our findings provide important novel structural insights for the design of multivalent FimH antagonists that bind with positive cooperativity.

FimH is the fimbrial adhesin from the Gram-negative *Escherichia coli* that accomplishes bacterial adhesion to mannosylated glycoproteins on the luminal side of epithelial linings (1). Subsequent invasion allows the bacteria to reach the basolateral side of the epithelium near the lamina propria (2). *E. coli* can induce IL-8 and CCL20 secretion by epithelial cells through the engagement of its flagellin with Toll-like receptor five, attracting neutrophils, and dendritic cells (3). Intracellular *E. coli* replicate in a large vacuole, inducing production of TNF-α and IL-12, which activates Th1 cells to produce IFN-γ (4). In response to TNF-α and IFN-γ stimulation and *E. coli* infection of intestinal epithelial cells, glycoprotein receptors for FimH are upregulated (5). This creates a loop of colonization and inflammation that can lead to chronic disease (6). FimH antagonists have been demonstrated not only to inhibit bacterial adhesion (7, 8) but also to provide an excellent anti-inflammatory therapy by lowering the secretion of proinflammatory cytokines IL-6, IL-12, IL-23, and IL-17 (9, 10). This explains the large potential of FimH as a therapeutic target for the treatment of Crohn’s disease (11, 12).

The lectin domain of the fimbrial adhesin FimH from *E. coli* recognizes with the highest affinity and in monovalent fashion oligomannose-3 and -5 *N*-glycans. This happens with a high affinity (*K*_d_ = 20 nM) as long as the α1,3-linked arm remains free from any further α1,2-mannose substitution. A latter substitution which generates the oligomannose-6 *N*-glycan causes a 10-fold loss in solution affinity between oligomannose-5 and oligomannose-6 (13). In this work, the binding of FimH to pauci- and oligomannose-containing *N*-glycans and equally glycosylated proteins, and all four possible dimannoside endings Manα1,2Man, Manα1,3Man, Manα1,4Man, and Manα1,6Man, have been probed using a glycan microarray (14) and the FimH LEctPROFILEplate assay (15, 16). The kinetics of binding, *k*_a_ and *k*_d_, have been analyzed using surface plasmon resonance (SPR) measurements and crystal structures and molecular dynamics (MD) simulations help to decipher the mechanism behind the monovalent and bivalent binding modes of oligomannose *N*-glycans to FimH.

Monovalent and divalent binding modes were found in cocrystal structures of the FimH lectin domain with core α1,6-fucosylated oligomannose-3 and with oligomannose-6, respectively. Core α1,6-fucosylation of oligomannose-3 does not change its binding through the α1,3-linked mannose arm. Conversely, oligomannose-6 binds to both mannoses on the α1,6-linked arm of the second trimannose core residing, instead of to a mannose linked to the common trimannose core of *N*-glycans. Manα1,2αMan, substituting the α1,3-linked arm on the primary trimannose core of oligomannose-6, was defined as a third possible FimH-binding epitope, however high association and dissociation rates of appear to disfavor the formation of a stable assembly *via* this nonreducing end (17).

Earlier reports on multivalent binding of natural oligomannose *N*-glycans measured using analytical gel filtration (18) demonstrated monovalent, bivalent, and trivalent complexes, similar to what has been evidenced for the interaction of FimH with the human urinary defense protein Tamm-Horsfall glycoprotein (THGP, also called uromodulin (19)), where THGP interacted multivalently with FimH (20). Here, we present a prime example of positive cooperativity in the bivalent binding of oligomannose-6 *N*-glycan by the bacterial FimH lectin. This binding occurs on the two glycan branches, different from the one found in monovalent interactions with FimH that carries the potential third binding site oligomannose-6 for FimH. Manα1,2Man, at the nonreducing end of the latter branch, is solvent accessible and positioned in between the Arg98 residues of the two bound FimH monomers but it is not occupied by FimH. The chitobiose moiety of oligomannose-6 lies in a groove on top of a junction between the two lectin domains and its reducing end is solvent accessible for eventual protein *N*-glycosylation.

## Results

### Glycan array screening with the FimH lectin

**Figure 1:**
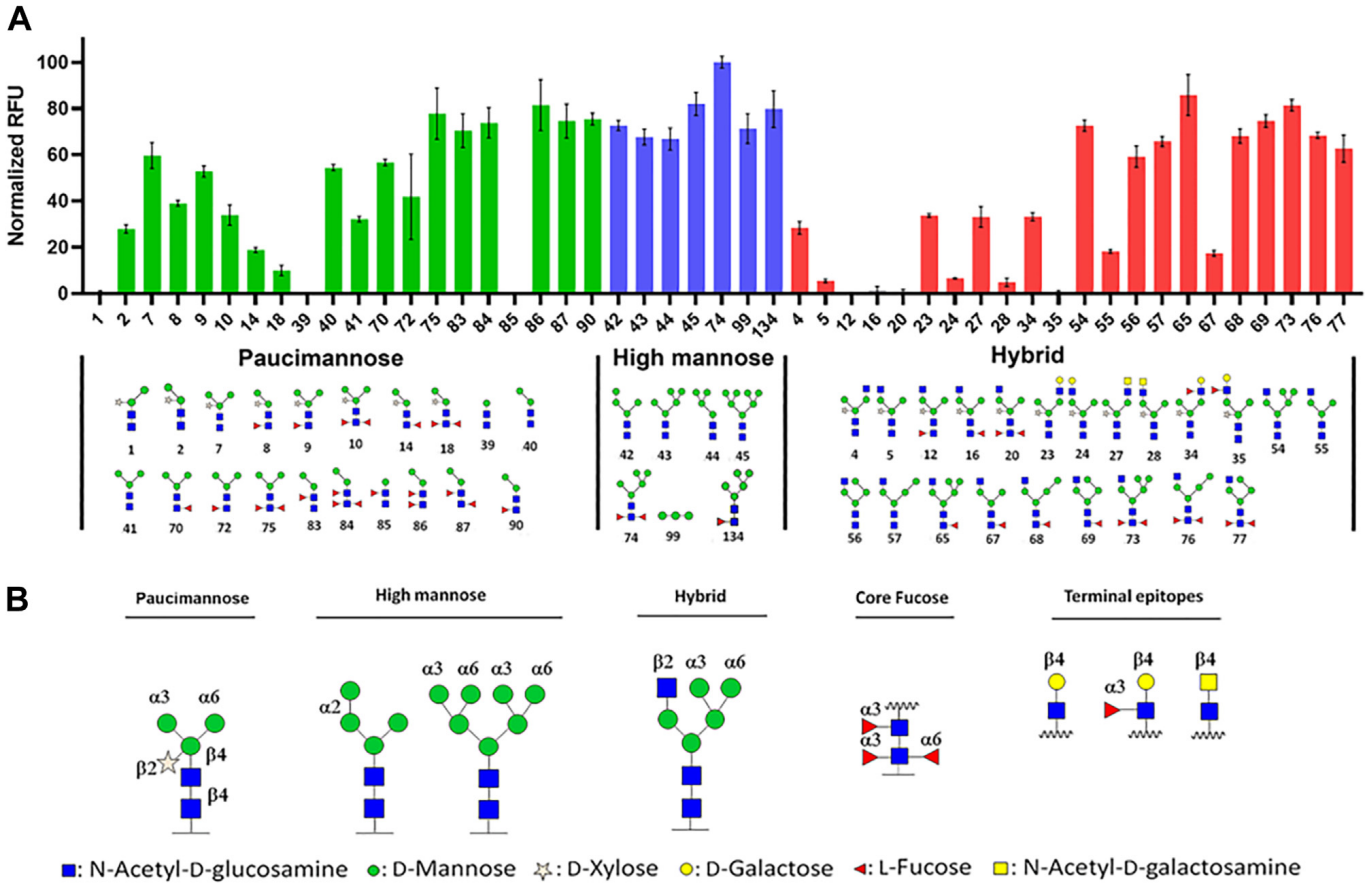
**Glycan microarray screening of FimH binding to paucimannose, high mannose, and hybrid-type *N*-glycans.***A*, glycan microarray incubation of FimH at 6.25 μg/ml concentration and binding was revealed using anti-FimH and anti-rabbit IgG-Alexa555. Each histogram represents the normalized relative fluorescence units **(**RFU) averaged from four spots along with the SD of the Mean. *B*, stereochemistry of the glycosidic bonds of the *N*-glycan structures represented.

The binding profile of the FimH lectin was studied toward a panel of synthetic *N*-glycan structures employing microarrays (Fig. S1). A solution of the lectin in binding buffer was incubated and the interactions detected with anti-FimH antibody and a fluorescently labeled secondary antibody. We could observe binding toward several mannose containing structures in the microarray (Fig. S2). Figure 1 summarizes the binding results toward paucimannose, high mannose and hybrid type *N*-glycans at a single protein concentration. From the data obtained with paucimannose structures, a clear preference for the terminal mannose in the α1,3 arm of the pentasaccharide core (Man3Gn2) of *N*-glycans could be observed, while FimH does not recognize efficiently the terminal mannose in the α1,6 arm on the common pentasaccharide *N*-glycan core (#1 *versus* #2). A similar pattern of recognition is observed among the hybrid *N*-glycans, with a nondetectable or very reduced binding when the only terminal mannose displayed is an α1,6-linked mannose. The binding of FimH at six different concentration (6.25, 12.5, 25, 50, 100, and 200 μg ml^−1^) allows to evaluate the dose response on this glycan array. Histograms for paucimannose, hybrid, and high mannose structures are shown separately (Fig. S2, *B*–*D*).

Among the paucimannose *N*-glycan structures, different substitutions in the *N*-glycan core such as chitobiose core fucosylation (up to three fucose residues) and the presence of β1,2 xylose interfere with, but do not abolish, the recognition of FimH on the glycan microarray. Observed relatively higher binding on the glycan array does not necessarily correlate well with previously reported solution affinities (13), which is most likely due to multivalency effects on the glycan array. Multivalency effects are very complex to evaluate because of their heterogenous nature (mixes of monovalent and multivalent interactions) and the high density of ligands on the glycan array surface, however the use of different concentrations of the analyte can help with the analysis (Fig. S2, *B*–*D*). It is reassuring that typical monovalent ligands at a molar excess of analyte on the array, such as Man3Gn2, #41 and Man2Gn2, #40, show a linear concentration-dependence on FimH.

Finally, the binding profile of FimH on glycan microarray is compared with those of the broadly studied mannose-binding lectins concanavalin A (ConA) and *Galanthus nivalis* agglutinin (GNA), in a dual color heat map (Fig. S3). FimH binding improves for glycans carrying extended linear epitopes Manα1,6Manα1,6Manβ1 (glycans #68, #76) over the shorter Manα1,6Manα1 branch (glycans #55, #67), whereas for the ConA plant lectin the affinity diminishes for the longer glycan. Such improvement was also found using solution affinity SPR by comparing dimannoside- and trimannoside-binding to FimH (13). GNA prefers terminal Manα1,3Man that is not in the context of a hybrid glycan.

### Monovalent binding of α1,6-core-fucosylated oligomannose-3 to the FimH lectin

**Figure 2:**
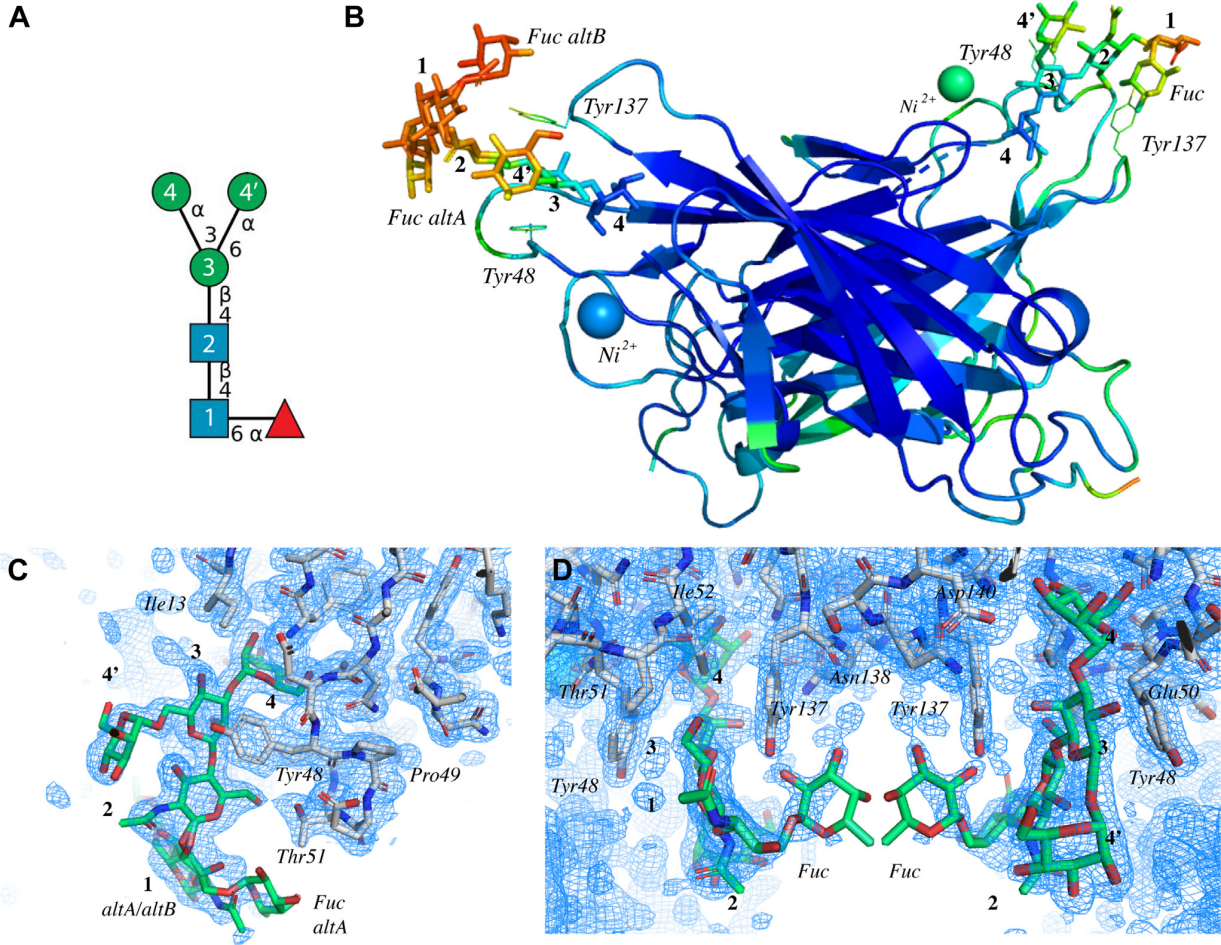
**Monovalent binding of the Man3Gn2F1[6] (*N*-glycan #70 in**Fig. 1**) ligand with FimH in the crystal (PDB entry****7BHD****).***A*, SNFG (60) presentation of Man3Gn2F1[6] using DrawGlycan-SNFG (53). *B*, the asymmetric unit content is color-coded according to the crystallographic temperature (B-) factors from *blue* (*cold*) over *green*, *yellow* and *orange* to *red* (*hot*). *C*, the electron density (2F_o_-F_c_ at 1.0 σ level) of Man3Gn2F1[6] in FimH chain A indicates the suggested position of the *altA* conformation of α1,6-fucosylated *N*-acetylglucosamine 1. *D*, the electron density (2F_o_-F_c_ at 1.0 σ level) for two core fucoses at a crystal lattice contact illustrates the suggested position of the *altB* conformation in FimH chain B. Figures *B*–*D* have been designed using Pymol (61). SNFG, Symbol Nomenclature for Glycans.

To visualize the interaction between FimH and core-fucosylated oligomannose-3, we have performed cocrystallization and obtained a crystal structure at 1.4 Å resolution (Table S1). The ligand Man3Gn2F1[6] interacts with FimH *via* its α1,3-arm, and the mannose binds in the monosaccharide-binding pocket (M) of FimH *via* the conserved ligand residues Phe1, Ile13, Asn46, Asp47, Tyr48, Ile52, Gln133, Asn135, Tyr137, and Asn140. The α1,6-fucosylated *N*-acetylglucosamine 1 in one of the FimH monomers of the asymmetric unit has a very high motility and was modeled in two alternate conformations, *altA* and *altB* (Fig. 2*B*). The *altB* binding mode is conserved over the two FimH monomers (Fig. 2, *C* and *D*).

We compared this new complex with the previously published crystal structure of the FimH–Man3Gn2 complex (PDB entry 2VCO (21)), to evaluate the effect of the addition of fucose. The two glycans, Man3Gn2F1[6] and Man3Gn2 respectively, interact with FimH *via* the Manα1,3Manβ1 disaccharide, oriented in the mannose-binding pocket of FimH in a similar way. The interaction created with Manα1,3Manβ1,4GlcNAc is therefore the same for the two ligands and allowed to maintain the potential hydrogen bond between O_6_ of GlcNAc 2 and the hydroxyl group of Thr51 (Fig. S4*C*). In one FimH monomer, the larger Man3Gn2F1[6] ligand samples more space, by rotating around the GlcNAc 1–GlcNAc 2 glycosidic bond (torsion angle ψ), resulting in at least two alternate conformations (Fig. 2*C*). On the other FimH monomer, two fucoses are close in the crystal packing and take on the *altB* conformation near the side chain of Tyr137 (Figs. 2*D* and S4*B*). Core fucosylation can influence the orientation of the chitobiose unit (GlcNAc 1 and GlcNAc 2 residues), that links the glycan to asparagine on a glycoprotein. Therefore, we wanted to understand the influence of core α1,6-fucosylation on the affinity of Man3Gn2 for FimH, why the Manα1,3Man arm was systematically chosen for binding by FimH and what would happen when this arm is further substituted as in the case of Man6Gn2.

### Core fucosylation of oligomannose-3 slows down association with FimH

**Figure 3:**
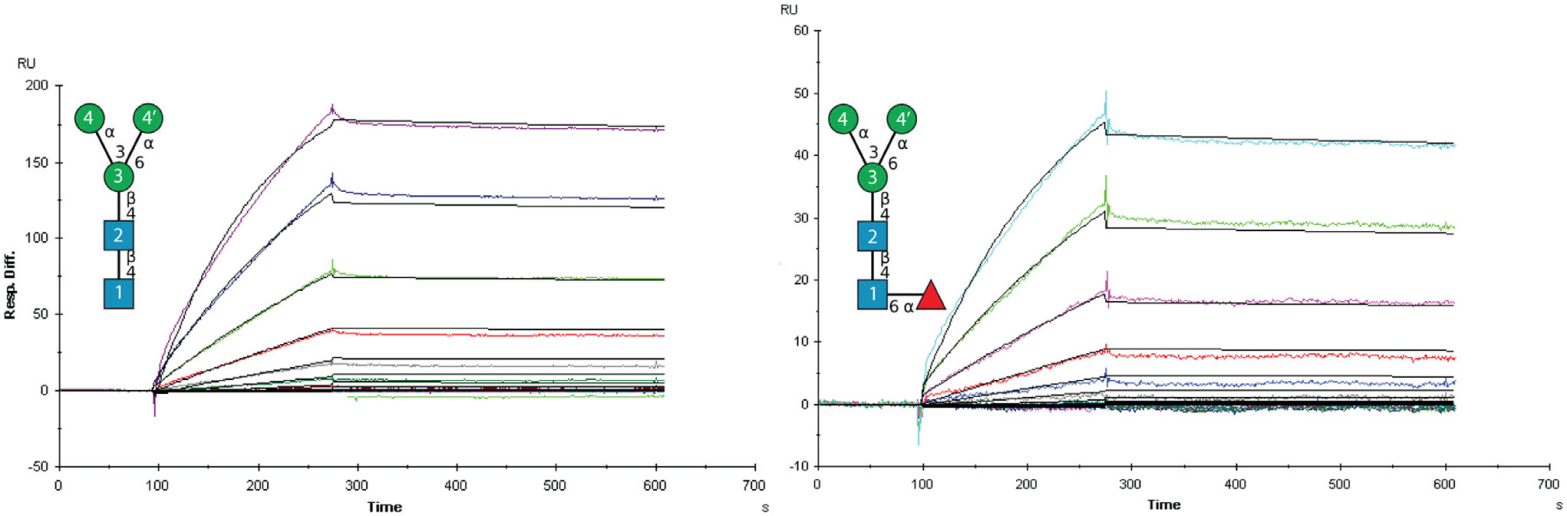
**SPR sensorgrams of the kinetics of binding of FimH, at concentrations (*colored curves*) between 0.13 μM and 8.58 μM, to immobilised Man3Gn2 and Man3Gn2F1[6] glycans (insets in SNFG symbols) and their fit (*black lines*) to the Langmuir 1:1 model.** SNFG, Symbol Nomenclature for Glycans; SPR, surface plasmon resonance.

To understand the influence of mammalian α1,6 core fucosylation on a paucimannosidic glycan in the interaction with FimH, the kinetic parameters with the oligomannose-3 glycans Man3Gn2 and Man3Gn2F1[6] have been measured using SPR detection (Fig. 3). Only small differences were observed with a preference of Man3Gn2 over Man3Gn2F1[6] (Table 1).

**Table 1:** SPR data for paucimannosidic structures as immobilized glycan ligands and on proteins

SPR variables and units	Imm. RU	R_max_ RU	SE (R_max_) RU	*k*_a_ M^−1^.s^−1^	SE (*k*_a_) M^−1^.s^−1^	*k*_d_ s^−1^	SE (*k*_d_) s^−1^	*K*_d_ nM	chi^2^
Glycan									
Man3Gn2	722	221.37	0.51	1073.76	3.8	7.82 10^−5^	6.00 10^−6^	72.8	6.65
Man3Gn2F1[6]	134	60.43	0.22	849.68	4.5	1.00 10^−4^	8.00 10^−7^	118	0.59
Glycoprotein ω1									
Man3Gn2	355	48.94	0.16	3890	22.58	1.30 10^−4^	2.39 10^−6^	33.6	1.27
Man3Gn2F1[6]	453	280.3	0.28	663	2.3	8.89 10^−5^	3.00 10^−7^	134	1.52
Man3Gn2F1[3]	468	303.3	0.31	525.4	0.91	6.72 10^−5^	2.97 10^−7^	128	1.46
X1Man3Gn2F1[3]	434	158.1	0.97	257	2.1	1.50 10^−3^	3.30 10^−6^	6000	0.263

Abbreviations: SE, standard error; SPR, surface plasmon resonance.

The higher affinity can be explained by a faster association and a slower dissociation of Man3Gn2 than for Man3Gn2F1[6] (Table 1). To compare the binding between FimH and these same *N*-glycans when present on glycoproteins, we have analyzed the kinetics of binding on the protein omega-1 (ω1), a major immunomodulatory *Schistosoma mansoni* soluble egg antigen that has been expressed in glycan-engineered *Nicotiana benthamiana* plants (22, 23). As we cannot regenerate the glycoprotein ligand on the chip due to its nanomolar affinity of FimH, we have applied the method of single cycle kinetics on a series of glycosylation forms of ω1 that involve paucimannosidic glycans (Fig. S5). The results of the fit to the Langmuir 1:1 binding model indicate a similar trend for the glycoprotein ligands as for the glycan ligands (Table 1).

The affinity (*K*_d_) is consistently lower, with the association rate (*k*_a_) being most hampered by core-fucosylation, both for the free glycan and conjugated to the protein (Table 1). Differences in dissociation rates (*k*_d_) of the paucimannosidic glycan Man3Gn2 with and without core fucosylation are minor but *k*_d_ is larger for core-fucosylated ligands. This is more clearly observed for the ω1-glycoproteins (Fig. S5). We notice, with these results, that the FimH has a submicromolar affinity for the differently glycosylated ω1-glycoproteins, except for the glycoprotein expressed in the WT plant carrying xylose β1,2-linked to the central mannose 3 of oligomannose-3 (Table 1 and Fig. S5). When xylose is still present, which is a prerequisite for α1,3-fucosylation to happen in plants, this most favorable interaction is hindered. Also, comparatively, FimH has 3-fold higher affinity for ω1-Man3Gn2 than for ω1-Man3Gn2F1[6] and ω1-Man3Gn2F1[3]. In conclusion, FimH has a better affinity for paucimannose-carrying ω1-glycoproteins devoid of xylose or fucose substitutions.

### FimH prefers Manα1,3Man over all other dimannosides

**Figure 4:**
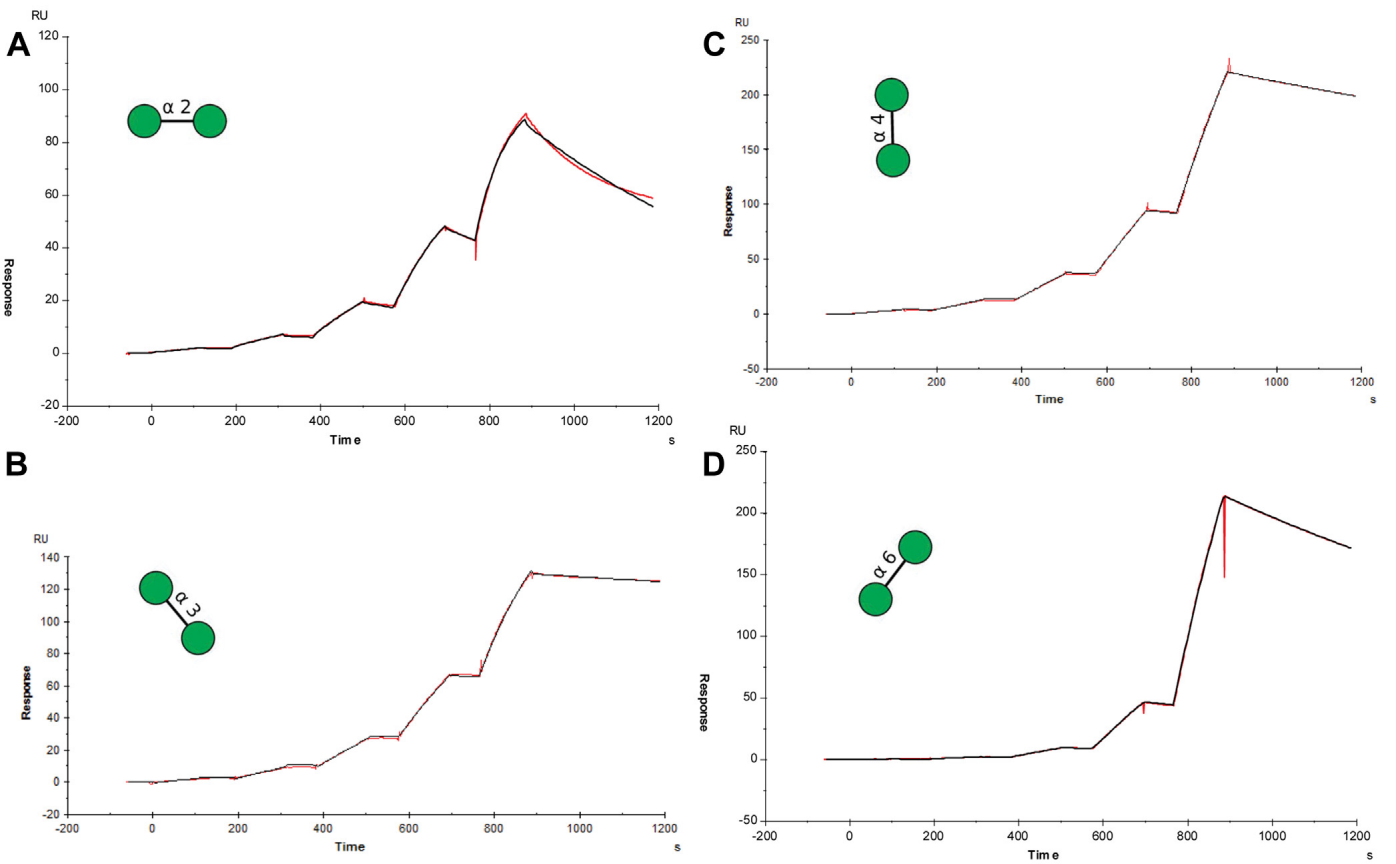
**Single cycle kinetics of FimH binding to immobilized BSA conjugates of the four possible α-dimannosides.***A*, Manα1,2Man-BSA. *B*, Manα1,3Man-BSA. *C*, Manα1,4Man-BSA. *D*, Manα1,6Man-BSA. BSA, bovine serum albumin.

The binding affinities of dimannoses toward the FimH lectin have been determined using SPR, Molecular Mechanics Poisson-Boltzmann Surface Area (MM-PBSA) calculations, and a lectin profile kit, respectively. Bovine serum albumin (BSA) conjugated to the four possible α-linked dimannosides have been immobilized on CM5 sensor chips, at approximately 100 RU each (Table 2), to present an equal number of BSA conjugates on the sensor surface in order to enable their comparison of kinetics of binding and of affinities (Fig. 4).

**Table 2:** Kinetic parameters (association and dissociation rates) for the binding of BSA-conjugated α-linked dimannosides to the FimH lectin domain, immobilized single or in equimolar (1:1) dual mixes

BSA-conjugated dimannose	Imm. RU	R_max_ RU	SE (R_max_) RU	*k*_a_ M^−1^.s^−1^	SE (*k*_a_) M^−1^.s^−1^	*k*_d_ s^−1^ (×10^−6^)	SE (*k*_d_) s^−1^ (×10^−6^)	K_d_ μM	Chi^2^
Manα1,2Man	86	107	0.1	219.8	0.8	1666	3.4	7.58	0.918
Manα1,3Man	108	210	2	332.6	7.7	124	4.8	0.372	0.928
Manα1,4Man	151	966	12	93.0	1.3	349	3.0	3.75	0.926
Manα1,6Man	161	1080	10	79.7	0.9	738	2.5	9.25	1.81
1:1 Manα1,2Man/Manα1,3Man	104	144	2	237.0	5.3	328	8.8	1.38	0.82
1:1 Manα1,3Man/Manα1,6Man	107	250	3	275.6	4.2	65.2	9.8	0.236	4.6
1:1 Manα1,2Man/Manα1,6Man	101	152	34	75.54	18	898	45	11.9	2.76

Abbreviations: BSA, bovine serum albumin; SE, standard error.

It is apparent from the single cycle kinetics experiment that the affinity of FimH ranges from the greatest for Manα1,3Man-BSA, much less for Manα1,4Man-BSA, further decreased for Manα1,2Man-BSA, and is least for Manα1,6Man-BSA (Fig. 4). This is due to a combination of the highest association rate and lowest dissociation rate for Manα1,3Man-BSA (Table 2). The latter thus displays the better affinity for FimH, with an at least 10-fold difference compared to the other BSA-dimannosides. Moreover, it is the only dimannoside displaying an affinity that is improved 3-fold over micromolar affinity, which is exactly in the same order of magnitude as the earlier molecular binding studies using solution affinity by SPR (13) and isothermal titration calorimetry (17).

**Figure 5:**
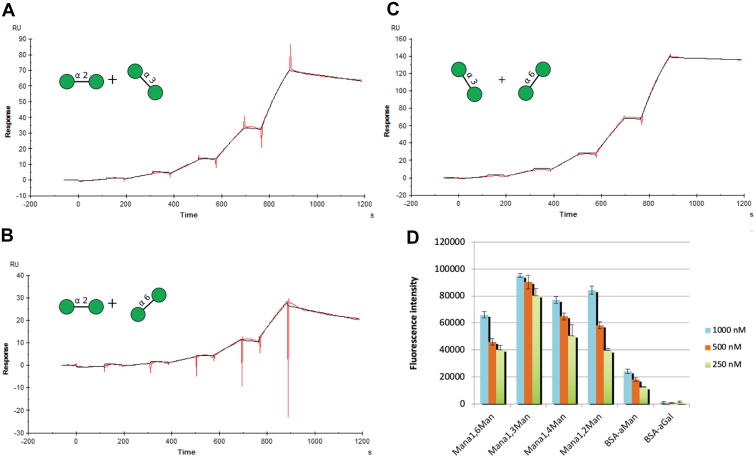
**Single-cycle kinetics and lectin profiling with FimH.***A*–*C*, SPR detection of FimH binding to equimolar mixes of α-linked dimannosides. *D*, direct binding of BSA-glycoconjugates to FimH in the LEctPROFILE plate assay. BSA, bovine serum albumin; SPR, surface plasmon resonance.

We designed a FimH LEctPROFILE kit and tested it to compare with the SPR results. FimH was fixed as a ligand on the microwell plate and different biotinylated BSA-dimannosides were analyzed at different concentrations and detected with fluorescently labeled streptavidin (Fig. 5*D*). With this result, we could again determine that among the different BSA-dimannoside glycoconjugates, FimH prefers to interact with Manα1,3Man, followed by Manα1,4Man, Manα1,2Man, and Manα1,6Man.

**Figure 6:**
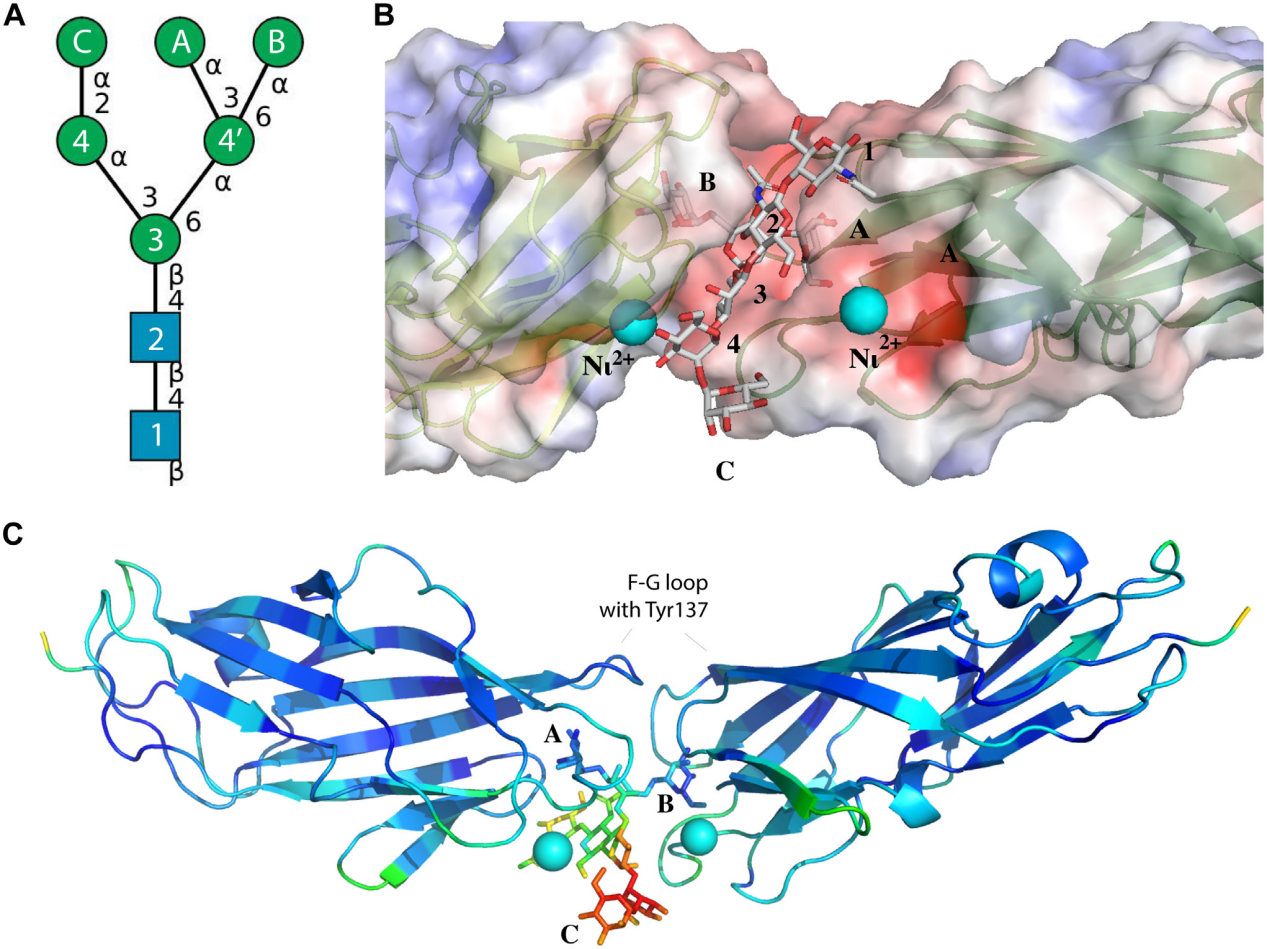
**Bivalent binding of FimH adhesin by oligomannose-6 in the crystal structure (PDB entry 7QUO).***A*, SNFG symbols, nomenclature, and linkage description of the oligomannose-6 *N*-glycan, here named Man6Gn2. *B*, a space-filling model allows a lateral look on the asymmetric plug-in of the A and B mannoses in the monosaccharide-binding pocket of two FimH lectin domains. The *cyan spheres* represent nickel ions, that were included at 10 mM in the crystallization condition. *C*, the averaged structure derived upon MD simulations illustrates a stable bivalent assembly. The cartoon is colored by the temperature (B-)factor, varying from *blue* (cold) over *green*, *yellow*, and *orange* to *red* (hot). MD, molecular dynamics; SNFG, Symbol Nomenclature for Glycans.

Next, we wanted to understand the behavior of FimH in terms of selectivity toward heterogenous dimannose populations, because they are as such presented on oligomannose-6 and on high-mannose type *N*-glycans (Fig. 6*A*). Therefore, we designed mixed surfaces by mixing pairs of BSA-dimannoses in a 1:1 M ratio and with a total immobilization rate of approximately 100 RU, similar as for the immobilization of unique mannoside (Table 2). In the kinetic titrations, performed with FimH lectin domain as the analyte, we observed a higher affinity for the mixed surfaces containing Manα1,3Man-BSA than when Manα1,3Man-BSA was not present in the equimolar mix (Fig. 5). The Manα1,3Man-BSA conjugate thereby dominated the kinetics of binding of FimH binding, as indicated by its high association and low dissociation rate. Moreover, a positive cooperativity was observed for the equimolar mixtures of Manα1,3Man-BSA and Manα1,6Man-BSA. This composition presented a one-and-a-half times lower dissociation rate *k*_d_, resulting in an increase in the affinity than for Manα1,3Man-BSA alone (Table 2).

MD simulations have been performed with the nonreducing α-d-mannose residue of each of the four different α-linked dimannoses and with α-d-mannose, docked in the monosaccharide-binding pocket. The region of the FimH lectin domain for the calculation of the binding energetics of the dimannose ligands was enlarged compared to earlier calculations (17) (Fig. S7), in order to prepare for MD simulations with larger oligomannoside structures. The energy contributions, computed from MD simulations using the enlarged region, are congruent with the experimental data as they validate a similar sequence in FimH dimannoside binding preferences (Tables 2 and S2). The only difference is an inversion of the order of preference between α1,4-linked and α1,2-linked dimannosides, most likely due to the large entropic contribution involved in Manα1,2Man binding (17) and the difficulty to take entropy into account in the calculations. The calculated Gibbs free energy change on binding is greatest for Manα1,3Man, followed by Manα1,2Man, Manα1,4Man, Manα1,6Man, and least for the monosaccharide Man (Table S2).

### Bivalent interactions of oligomannose-6 with FimH

Oligomannose-6 (Man6Gn2) *N*-glycan in complex with FimH lectin domain cocrystallised on top of a large salt crystal (Fig. S6). It binds bivalently to FimH in the crystal structure *via* two of its branches carrying the mannoses A and B (Fig. 6). Mannoses A and B are two nonreducing end residues of the Manα1,3Manα1 and Manα1,6Manα1 branches, respectively, where the shared reducing end mannose is the central mannose 4′. Together they form a second trimannose core (A-4′-B) that is α1,6-linked to mannose 3 of oligomannose-3 (Fig. 6*A*). One FimH lectin binds the α1,6-linked mannose of the third arm, Manα-1,6Manα-1,6 Manβ-1 (B-4′-3), in its monosaccharide-binding pocket, whereas a second FimH lectin anchors the α1,3-linked mannose of the middle arm Manα1,3Manα1,6Manβ1 (A-4′-3) (Fig. 6*B*). The central mannose 4′ hinges between the two FimH lectin domains. This bivalent assembly as found in the crystal structure, remains stable during a 100-ns MD simulation, without the terminal mannoses A or B attempting to reorient or to leave their respective monosaccharide binding pocket (Fig. 6*C*).

**Figure 7:**
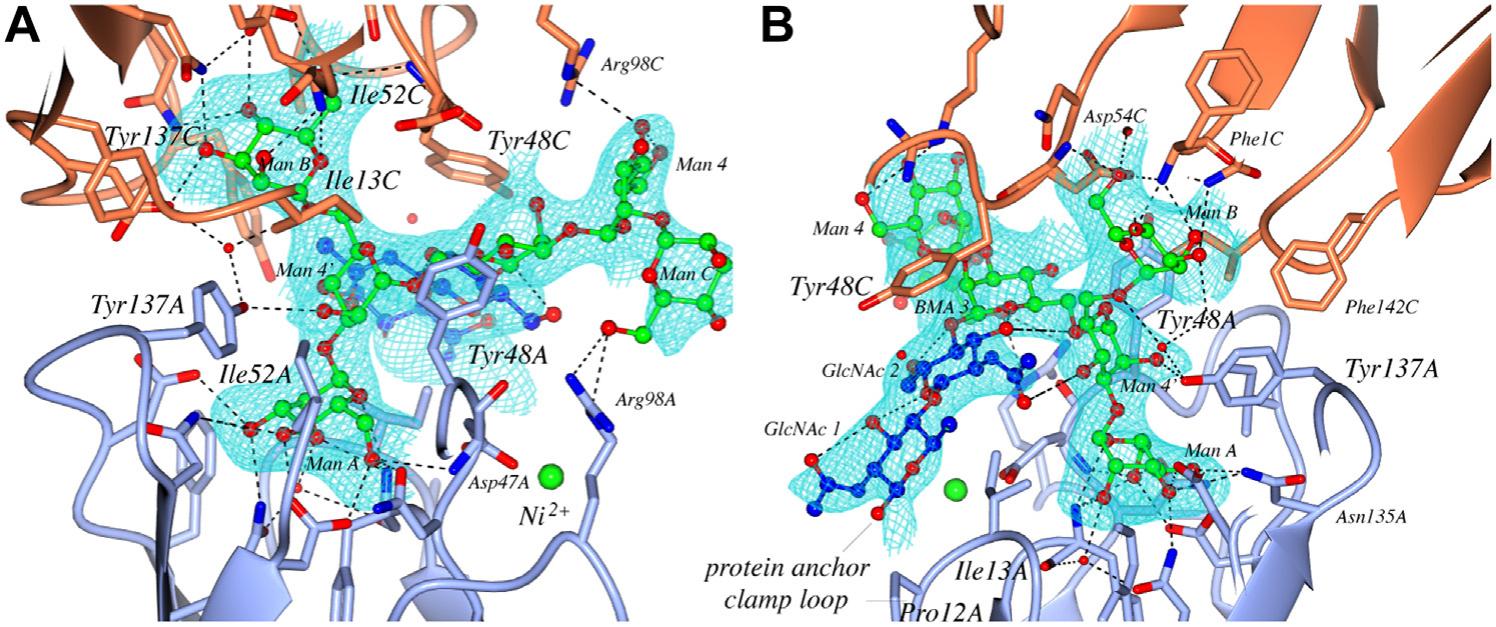
**Bivalent interactions involving two FimH lectins made by oligomannose-6.***A*, the nonreducing A and B mannoses are connected by a central mannose 4′ in a second trimannose core (A-4′-B). All four oligomannose-6 branches are supported by an asymmetric tetrahedron formed between tyrosine residues 48 and 137. *B*, 180° rotation around a vertical axis, relative to A: mannose 4′ is α1,6-linked to β-d-mannose 3, the central residue in the trimannose core (4-3-4′) common to *N*-glycans on glycoproteins. The chitobiose moiety is hovering over the clamp loop characterised by Ile13. Figures prepared using CCP4mg (62), with omit maps for the glycan ligands generated in Privateer (45).

The crystal structure shows that the Man6Gn2 ligand pulls the two bound FimH lectin domains closely together head-to-head, *via* the binding to the nonreducing end mannoses A and B. The FimH lectin further assemble head-to-tail to form a concatenate in the asymmetric unit of crystal. The head-to-head assembly was shown earlier to be as plausible as head-to-tail, in heptyl α-d-mannose ligand-induced oligomerization of FimH (24). A suture of the protein surface can be remarked behind the central mannose 4′ (Fig. 6*B*). The chitobiose moiety (residues GlcNAc 2–GlcNAc 1) and the Manα1,2Manα1,3Manβ1 (mannose residues C-4-3) arm ly in the groove resulting from the suture. These other two glycan branches of oligomannose-6 are also strongly involved in interactions with FimH, although not through its monosaccharide-binding site but through the tyrosine gate residues Tyr48 and Tyr137, their mediator residue Ile52 (25), as well as by Ile13 included in the clamp loop (26) (Fig. 7). It can be seen, nonetheless, that these branches are the most flexible and sprout out from the open front (Fig. 6). Their termini are the most solvent exposed, which would be needed for the GlcNAc 1 residue in order to make an *N*-glycosidic linkage to asparagine of a protein and for mannose C to be a third FimH-binding epitope in the formation of trivalent complexes (18).

In the bivalent binding by the Man6Gn2 *N*-glycan, the protein-protein interface is strongly interwoven. Both the Tyr137 residues, located in the loop between β-strands F and G (F–G loop, Fig. 6*C*) adopt a conformation that strengthens the aggregation of FimH around the glycan ligand. In the interface, the tyrosine gate residues Tyr48 and Tyr137 form a tetrahedron, a most economic space-filling structure, at the boundary of the asymmetrically bound *N*-glycan, with each tyrosine side chain stacking against a saccharide residue (Fig. 7). In the FimH monomer with mannose A (ManA) buried in the monosaccharide-binding pocket (chain A), Tyr48A lines up next to the central α-mannose 4′ and the Tyr137A hydroxyl group makes a hydrogen bond with O_2_ of that same mannose residue (Fig. 7*A*). In the second FimH lectin that buries mannose C (Man B) in the monosaccharide-binding pocket (chain C), Tyr48C stacks parallel to the GlcNAc 2 ring (Fig. 7*B*). The rather flat chitobiose moiety lies between the two lectin domains, sandwiched between Pro12A-Ile13A on one side and Tyr48C-Thr51C on the other side (Table S4). Tyr137C tops off the reducing end branch in a hydrophobic contact with the *N*-acetyl group of GlcNAc 2. Arg98 is the most important residue surrounding almost symmetrically the mannose C residue (Fig. 7*A*).

### Switch from monovalent to bivalent *N*-glycan binding

With the crystal structure available for an *N*-glycan binding bivalently to FimH, a structural and functional analysis in order to decipher the driving forces favoring bivalent *versus* monovalent *N*-glycan binding becomes imminent. We compared the two new crystal structures with crystal structures of FimH in complex with Man3Gn2 (PDB entry 2VCO) and with Man3 (PDB entry 6GTV), in terms of their protein–carbohydrate interactions (Table S4). We computed the free energy of binding, ΔG, for each of the different glycans, using a hybrid MM-PBSA approach (Table S3), identical to what had been performed for the dimannoside glycans.

MD simulations were performed with oligomannosides of growing complexity, using either the crystal structure of the complex or docking (Table S3). These simulations clearly highlight that Manα1,3Man at the nonreducing end is always preferred over any other glycan arm, which agrees with the observations for the dimannoside series (Table S2). Interestingly, the difference in free energy of binding between the two binding nonreducing mannose ends A and B is relatively smaller for those glycan structures containing the second trimannose core (A-4′-B) (Table S3). In the crystal structure of donor-strand complemented, two-domain adhesin, FimH with trimannose (Man3) (PDB entry 6GTV), the bivalently bound trimannose is positioned in the same way as its moiety in Man6Gn2 *N*-glycan, however it is enrobed inside a hollow cylinder pulled up by the tyrosine gate (48 and 137) and isoleucine (13 and 52) residues from the two interfacing FimH monosaccharide(M)-binding pockets (PDB entry 6GTW) (18). The chitobiose moiety of Man6Gn2 requires the hollow cylindrical interface to open at the reducing end (the central mannose) of Man3, in order to disengage the Tyr48 side chains that are essential for stacking interactions beyond M + 1 in the FimH–Man6Gn2 complex (Fig. 7 and Table S4). This opening up is already visible in the FimH–Man3 crystal structure containing only the lectin domain (PDB entry 6GTW).

**Figure 8:**
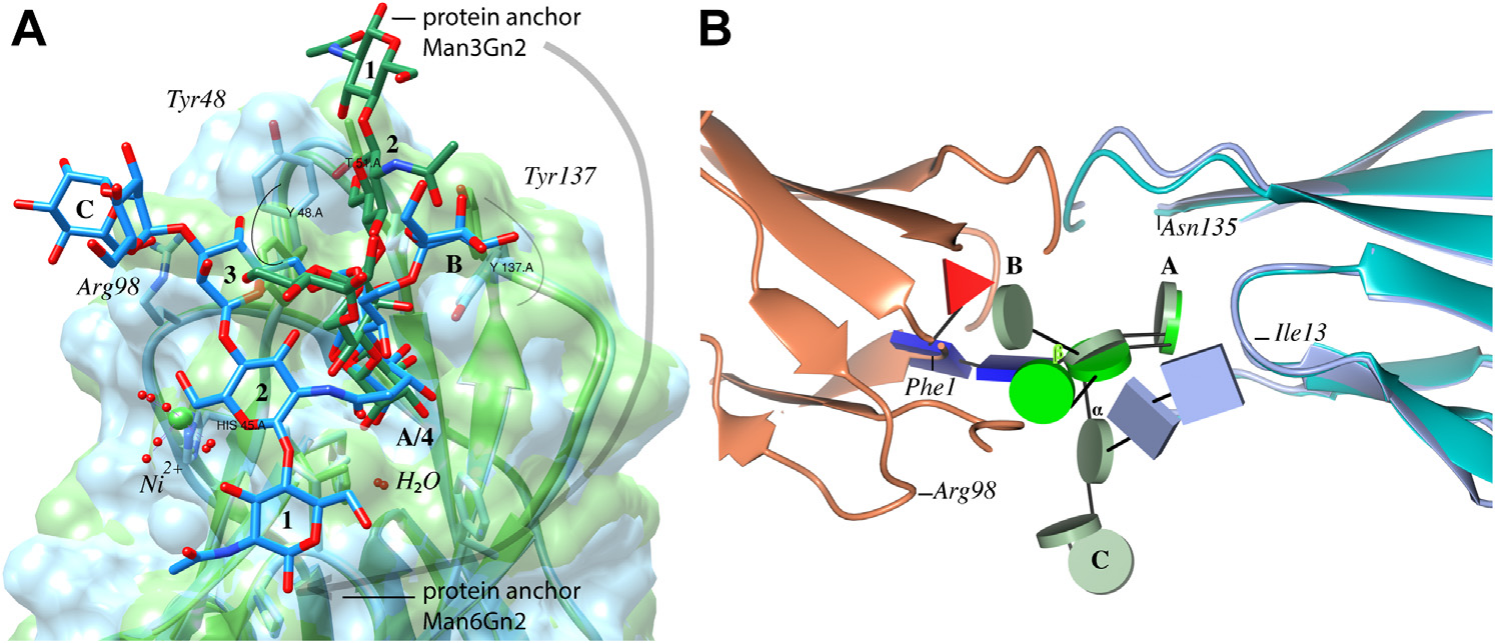
**Superposition of the crystal structures of FimH complexed with oligomannose-6 or oligomannose-3 glycans.***A*, mannose A of Man6Gn2 (PDB entry 7QUO, *pastel blue*) superimposes on mannose 4 of Man3Gn2 (PDB entry 2VCO, *pastel green*) in the monosaccharide-binding pocket of FimH. Figure created using Chimera (63). *B*, the bivalent assembly of Man6Gn2 (*pastel colors*) with two FimH lectin domains superimposed on the monovalent complex of FimH (*aqua*) with Man3Gn2F1[6] (SNFG colors) (PDB entry 7BHD) again with mannoses A and four overlapping. Figure *B* was generated using Glycoblocks (64) in CCP4mg (62).

Perhaps the most striking observation is the impossibility of oligomannose-6 to bind where Tyr48 is in an open-gate conformation: the central α-mannose 4′ and the central β-mannose 3 notably travel through the same space (Fig. 8*A*). Binding of the Manα1,6Manβ1 of Man3Gn2 would cause a collision with Tyr48A in its open gate conformation. The Tyr48A side chain adopts the closed gate conformation alike in the FimH bound to dimannoside Manα1,6Manα1-OMe and to the Manα1,6Manα1,6Manβ1 arm in oligomannose-6 (Fig. S8). As Tyr48A takes on the closed gate conformation to allow the passage of mannose 4′, it loses the carbohydrate ring-aromatic ring stacking typifying the binding of β-mannose 3 in Man3Gn2 (21) (Fig. S4) and in Man3Gn2F1[6] (Fig. 2, *C* and *D*). Remarkably, the aromatic side chain of Tyr48C salvages this loss in a parallel stacking with GlcNAc 2 of Man6Gn2 (Fig. 7*B*). Whilst the Tyr48A side chain moves to free the path for the transit of mannoses 4′ and 3, the Tyr137A side chain moves and establishes a potential hydrogen bond, *via* its side chain hydroxyl with the axial hydroxyl group on C_2_ of mannose 4’ (Figs. 7*A* and 8*A*). Tyr137C makes a potential hydrogen bond with the hydroxyl group on C_6_ of GlcNAc 1 (Table S4), thereby stabilizing the outward orientation of chitobiose. The solvent accessibility of the reducing end GlcNAc 1 would allow for conjugation to a protein by *N*-glycosylation. Interestingly, because the orientation of the chitobiose moiety has changed with 180° compared to in the monovalent complex with Man3Gn2, the relative position of the bound glycoprotein will also change (Fig. 8). It remains to be seen whether this may possibly provoke altered cell signaling, for example upon *E. coli* type-1 fimbrial adhesion (27).

The second FimH lectin binds Manα1,6Manα (B-4′) in the same site that had been occupied by the chitobiose (2-1) in the monovalent complex. When comparing to Man3Gn2F1[6], mannose B comes in nearly the same place as alternate conformation B of fucose, being the position of the core fucose that is conserved between the two Man3Gn1F1[6] protein chains (Fig. 8*B*). Mannose 4′ is less interactive (Table S4), as it mainly plays the role of the hinge between the two monosaccharide-binding pockets occupied by mannoses A and B, respectively. In this way, suppleness is permitted in the relative orientations of the bridged FimH lectin domains, a characteristic that has also been observed earlier in the crystals of FimH–trimannose (Man3) complexes (PDB entries 6GTV and 6GTW (18)).

## Discussion

Hereby, we describe the conversion from monovalent- to bivalent *N*-glycan binding to the FimH lectin domain, with two full *N*-glycan structures, namely α1,6-fucosylated oligomannose-3 (Man3Gn2F1[6]) and oligomannose-6 (Man6Gn2). Multivalent interactions by binding two or three FimH adhesins with high-mannose type *N*-glycans have a high biological relevance as they are molecular interactions that occur when the fimbrial adhesin binds to natural glycoprotein receptors, such as uroplakin 1a and THGP (19), on or near epithelial surfaces for *E. coli* colonization. Other *in vitro* studies on oligomannose–FimH interactions have demonstrated bivalent and trivalent binding of FimH using analytical gel filtration (18). We show that the interaction with oligomannose-6 experiences a true multivalency effect, notably by showing that a bivalent complex is formed that is more stable than the monovalent interaction with Manα1,3Man (the A-4′ dimannose) alone. The bivalent interaction occurs with a positive cooperativity, as demonstrated by measuring the kinetics of binding (Table 2 and Fig. 5) and by MD simulations (Table S3). Positive cooperativity has still been very rarely demonstrated but indicates that the interaction with a multivalent natural ligand, being the oligomannoside-6 *N*-glycan, has the molecular design that is favorable to generate multivalent FimH antagonists.

The preference of FimH is markedly selective for the α1,3-linked mannose branch of α1,6 core-fucosylated oligomannose-3, identical to what was found for oligomannose-3 without fucose (Fig. 2). Oligomannose-6, on the other hand, binds bivalently by engaging the α1,3-linked (A) and α1,6-linked (B) nonreducing mannoses in binding in the monosaccharide (M)-binding pocket of FimH (Fig. 6). Thus, while Man3Gn2 *N*-glycans, core-fucosylated or not, bind in a monovalent fashion in the crystal, Man6Gn2 bound to two FimH lectin domains (Fig. 7). Nonetheless, according to an earlier study using analytical gel filtration (18), Man3Gn2 and Man5Gn2 can also bind divalently, albeit with a negative cooperativity caused by the chitobiose (GlcNAc residues 1 and 2), and limited to the condition where the molar concentration of the oligomannose ligand does not exceed half the molar concentration of FimH. The equimolar presence of Man3Gn2F1(6) and FimH in our work does not correspond to the right conditions to observe bivalent binding. Bivalent binding was observed for Man3Gn2 and Man5Gn2 but this type of complexes had completely vanished at a 1.0:1.0 M ratio (18). Surprisingly, for Man6Gn2, this ratio could be easily exceeded while maintaining a good percentage of bivalently bound *N*-glycan (18). In the simulations of the expected gel filtration profile (18), bivalent binding of Man6Gn2 was seen to start before a 0.5:1.0 M ratio was reached and held on beyond, in contrast to for Man5Gn2 where divalent binding is completely abolished even before an equimolar ratio of FimH:Man5Gn2 was reached. Man6Gn2 was also the only *N*-glycan showing more bi-than monovalent binding at the 1.0:2.0 M ratio, contrary to Man3Gn2 and Man5Gn2. Interestingly, the same characteristics as for Man6Gn2 held true for structures lacking the chitobiose, Man3 and Man5 (18). The molar excess of the Man6Gn2 glycan (1 mM) over FimH (0.8 mM) in the cocrystallization condition is such that not more than 30% of the protein should bind bivalently, according to the results from analytical gel filtration (18).The stabilizing effect of cooperative bivalent binding may also explain why Man6Gn2 is bound to two FimH lectin domains in the crystals.

Finally, it has previously been reported that Man6Gn2 can even bind trivalently to FimH, although the latter never exceeded 18% (18). Structural data of a trivalent cluster of FimH on a ligand do not yet exist, other than on a β-cyclodextrin decorated with heptyl α-d-mannoside, acquired by means of small-angle X-ray solution scattering (28). We could easily observe the third potential binding site on mannose C of Man6Gn2 in our crystal structure. Although the site is solvent accessible, it is not occupied by a third FimH lectin domain. It makes contacts with Arg98 (Table S4) facing the nickel-binding site (Figure 6, Figure 7, Figure 8). Manual docking indicates that the glycan arm is not long enough to bury mannose C deep into the monosaccharide-binding pocket, hence the FimH protein is sterically hindered. Completing this glycan branch with another mannose, α1,2-linked to mannose C, or identical to isomer 7D1 (one of the Man7Gn2 isomers), might potentially increase the likelihood of a trivalent binding. A similar case is found for a lectin from the cyanobacterium *Nostoc ellipsosporum*. Cyanovirin-N binds Man6Gn2 using the third epitope for FimH and achieves nanomolar affinity for Man9Gn2 upon completion of the branch with another α1,2-linked mannose D1 (29).

In the bivalent assembly of FimH with oligomannose-6, the nonreducing mannoses A and B of Man6Gn2 are linked with the central mannose 4′, *via* an α1,3 and an α1,6 glycosidic bond respectively, to form a second trimannose core, A-4′-B (Fig. 6*A*). It was not previously noted that the central mannose of Man3 (Manα1,3(Manα1,6)Manα/β1) spontaneously adopts the α-anomeric configuration in its bivalent complex with FimH (PDB entries 6GTW for the lectin domain only and 6GTV for the donor-strand complemented full-length FimH) (18). Man3 makes similar interactions in the monosaccharide-binding pocket M of FimH, with mannose 4′ in the M + 1 site forming a hinge between the mannoses A and B (Table S4). In other words, the trimannose (A-4′-B) structure is present both in Man6Gn2 and Man3 that share this same binding epitope for FimH. It differs from the common trimannose core (4-3-4′) in that the central, reducing mannose is restricted to an α-anomeric configuration, instead of a β-anomeric configuration. Interestingly, in the complex of Man3 bivalently bound to the *Burkholderia cenocepacia* soluble lectin BC2L-A, only the α-anomeric configuration of the central mannose was retained (PDB entry 2WRA) (30). Therefore, it is identical to the second trimannose core, A-4′-B, of *N*-glycans. Man3 binds bidirectionally to BC2L-A, where each binding site holds 50% of mannoside A and 50% of mannoside B. This is repeated by the crystal lattice, creating a lectin filament. *Pterocarpus angolensis* seed lectin uses the same second trimannose core as BC2L-A for its interaction. The binding site of this lectin expands as the glycan structure extends from Man6Gn2 to Man9Gn2 but the interactions with the central α-d-mannose 4′ are consistently maintained (31). Only monovalent binding has been observed, where the lectin anchor mannose units inner to an oligomannose sequence. This is very different from bivalent *N*-glycan binding to FimH that exclusively anchors linear epitopes of a glycan arm by the terminal mannose in its monosaccharide-binding pocket (M) and where high-mannose type *N*-glycans can cluster multiple FimH lectins (32).

All high mannose N-glycans present on the glycan array show a very important recognition by FimH, which complicates the finding of specific binding patterns. Unfortunately, oligomannose-6 was not available for printing on the array. Nevertheless, we can observe that FimH largely disregards the α1,6-linked mannose when it is the sole mannose at the nonreducing end of a paucimannose or hybrid-type structure (Figs. 1 and S2*A*). The recognition by FimH improves when a second α1,6-linked mannose is present as a part of the second trimannose core (A-4′-B), in the sequence Manα1,6Manα1,6Manβ1. Such a linear sequence is present in Man6Gn2 and in Man5Gn2. Man5Gn2 bound to human THGP was recently shown in a monovalent complex with FimH, in a structure obtained by cryo-CM (PDB entry 7Q3N (33)). The same high-affinity epitope as in Man3Gn2, Manα1,3Manβ1GlcNAcβ1,4GlcNAcβ1, was recognized, again illustrating that the presence of the second trimannose core in Man5Gn2 on its own is not sufficient to make the switch to bivalent binding.

The binding profile of FimH was compared with two well-characterized mannose binding plant lectins ConA and GNA in a heat map (Fig. S3). Although the three lectins showed a very similar, mannose-binding profile, FimH efficiently recognized paucimannose *N*-glycans substituted with a core α1,3-fucose on GlcNAc 2 of the chitobiose core, while both ConA and GNA do not recognize these structures. That is interesting, because the crystal structure of FimH in complex with Man3Gn2F1[6] confirmed previous data on Man3Gn2 binding, in that the chitobiose moiety (GlcNAc 2–GlcNAc 1) gets priority over Manα1,6Manβ1 (4′-3) for interactions with FimH in the monovalent binding mode (Fig. 2) in their competition for the same space (Fig. 8). Chitobiose is required as part of the high-affinity epitope of Man3Gn2 (13, 21). The switch from monovalent to bivalent binding goes together with the chitobiose (2-1) tumbling over 180° into a new site created by the protein-protein interface (Fig. 8). This is probably why monovalent binding is observed in FimH–oligomannose-3 complexes (21) and why the nonreducing Manα1,6Manα end can only conquer this site once the high-affinity binding epitope mannose 4 is shielded by Manα1,2Man (34).

MD simulations of the FimH lectin with the three linked dimannoside endings supported that it is not only the interaction energy of the individual monovalent binding to FimH that determines the finally retained complex. We measured a positive cooperativity between mannoses A and B of Man6Gn2, based on the kinetics of FimH binding to mixed sensor interfaces of Manα1,3Manα1-BSA and Manα1,6Manα1-BSA (Fig. 5). However, this experiment did not consider dynamical and spatial restraints of the asymmetric bivalent complex. In conclusion, we presented two new crystal structures of natural oligomannose *N*-glycans bound to FimH, demonstrating how kinetics and stability of the complexes drive the most favorable arrangement between the *N*-glycan and the lectin in the crystal, either in a monovalent or bivalent interaction mode and on different *N*-glycan branches. We also determined the third mannose binding site for FimH on oligomannose-6 on the *N*-glycan branch carrying mannose C, in an environment dominated by charged interactions with Arg98 and the nickel ion-binding site (Fig. 8). The availability of a crystal structures of a complex of FimH with a natural bivalent *N*-glycan allows to better understand the molecular principles and rules of selectivity of FimH for *N*-glycan branches in the making and breaking of monovalent, bivalent, and trivalent recognition events of glycoproteins. These new mechanistic insights can help to progress the design of multivalent FimH antagonist glycomimetics to prevent colonization by *E. coli*. Multivalent inhibitors are needed to efficiently compete with the Velcro-like adherence of *E. coli* that uses multiple type-1 pili, carrying the FimH lectin at their tip, simultaneously to bind glycoproteins such as uroplakin 1a (35), uromodulin (33) and CEACAM6 (5) on epithelial cell linings.

## Experimental procedures

### FimH protein and oligomannosides

The FimH lectin domain (residues Phe1-Thr158) was expressed from a pET24a vector in the *E. coli* C43(DE3) expression strain (36) and purified as described earlier (21) in a single step on an sulfopropyl fast flow cation exchange chromatography in a 20 mM formic acid buffer at pH 4.0, before being dialyzed against 20 mM Hepes at pH 7.4 with 150 mM NaCl. Man3Gn2F1[6] or α1,6 core-fucosylated oligomannose-3 was obtained in large enough amounts for cocrystallization with FimH through chemical synthesis (14) (Figs. S9 and S10). The oligomannose-6 (Man6Gn2), and BSA modified with the Manα1,2Man, Manα1,3Man, Manα1,4Man, or Manα1,6Man dimannosides were all purchased from Dextra Laboratories.

### Glycan microarray ligand screening

The glycan microarrays were prepared as described (37). Briefly, 50 μM ligand solutions (1.25 nl, 5 drops, 250 pL drop volume) in sodium phosphate buffer (300 mM, 0.005% Tween-20, pH = 8.4) were spatially arrayed employing a robotic noncontact piezoelectric spotter (SciFLEXARRAYER S11, Scienion) onto *N*-hydroxysuccinimide (NHS) activated glass slides (Nexterion H, Schott AG). After printing, the slides were placed in a 75% humidity chamber for 18 h at 25 °C. The remaining NHS groups were quenched with 50 mM solution of ethanolamine in sodium borate buffer (50 mM, pH = 9.0) for 1 h. The slides were washed with PBST (PBS/0.05% Tween-20), PBS and water, then dried in a slide spinner and stored at −20 °C until use. FimH protein was diluted to 6.25, 12.5, and 25 μg/ml in PBS (1% BSA, 0.01% Tween-20). The polyclonal anti-FimH antibody solution (1: 1000 dilution, 200 μl) was incubated on the microarrays for 1 h at RT. The slides were washed with PBST. Next, they were incubated with Alexa Fluor 555 Goat Anti-rabbit IgG(1:1000) (Thermo Fischer Scientific) in PBS (1% BSA, 0.01% Tween-20) for 1 h in the dark. The microarrays were washed to remove unbound antibody with PBST, PBS and water, and subsequently dried in a slide spinner. The fluorescence measurements were performed on Agilent G2565BA Microarray Scanner (Agilent Technologies) at 10 μm resolution. The quantification of fluorescence was carried out using ProScanArray Express software (PerkinElmer; https://www.perkinelmer.com/uk/lab-products-and-services/resources/software-downloads.html) employing an adaptive circle quantification method from 50 μm (minimum spot diameter) to 300 μm (maximum spot diameter). Average relative fluorescence units values with local background subtraction of four spots and SD of the mean were reported using Microsoft Excel and GraphPad Prism software (https://www.graphpad.com/features). Maximum intensity relative fluorescence units normalization was applied and is presented in Figure 1.

### Crystallization and data collection on FimH-Man3Gn2F1[6]

FimH lectin domain was concentrated to 17 mg/ml, equal to 1 mM of protein, and mixed with 1 mM of M3Gn2F1[6]. The cocrystallization was set up at 20 °C using the sitting-drop vapor diffusion method. Crystals appeared in 1.1 M Li_2_SO_4_, 0.1 M Tris–HCl at pH = 9.0, 0.01 M NiCl_2_, and 3% glycerol. Data were collected at beamline Proxima-1 of the Synchrotron Soleil in Saint-Aubin, France. The crystals diffracted to 1.4 Å resolution. PDB entry 2VCO (21) was used as a model, upon removal of the *N*-glycan ligand Man3Gn2 and water molecules, to resolve the crystal structure using molecular replacement using PHASER (38). Crystallographic refinement was performed using phenix.refine (39) from the Phenix package (40) and the refined model was manually adjusted using the graphics program Coot (41, 42, 43) (Table S1). Model refinement was finalized using Refmac5 (44) and the *N*-glycan ligands have been validated using Privateer (45). These steps have been repeated using data that were rescaled and remerged using Staraniso (46, 47), in order to account for anisotropy in the diffraction data (Table S1).

### Crystallization and data collection on FimH-Man6Gn2

FimH lectin was concentrated to 13.6 mg/ml (0.8 mM) and mixed with 1 mM of the Man6Gn2. The cocrystallization was set up at 18 °C using the hanging drop vapor diffusion method. Crystals appeared in 1.0 M Li_2_SO_4_, 0.1 M Tris–HCl at pH = 8.5, 0.01 M NiCl_2_, and 3% glycerol. Data were collected to 3.0 Ǻ resolution at beamline P14 of the Synchrotron Petra III in Hamburg, Germany, in a serial crystallography approach upon locating five crystals grown onto the surface of a large salt crystal (Fig. S6) using a grid scan (48, 49). Molecular replacement was performed using FimH with PDB code 5FX3 (25). The structure was refined using Phenix (40) and finalized using Refmac5 (44). The glycan structure torsion library was corrected (50) and validated using Privateer (45). These steps have been repeated using data that were rescaled and remerged using Staraniso (46, 47), in order to account for anisotropy in the diffraction data (Table S1).

### Induced-fit docking of the oligomannosides

Different dimannosides and oligomannosides were docked into the FimH-binding site using the GOLD software (The Cambridge Crystallographic Data Centre; https://www.ccdc.cam.ac.uk/solutions/software/gold/). The six carbon atoms of the mannose ring of the ligand HM found in the coordinate file (PDB entry 4BUQ (51)) were used as a scaffold in the active site. A single internal structural water (below the axial O_2_ hydroxyl group of the mannose ring of Man3Gn2) in the active site was treated explicitly. The FimH lectin domain cocrystallized with Man3Gn2 (PDB entry 2VCO (21)) was used as the starting point protein conformation. The side chains of ten residues interacting with the mannose of HM in the binding site: Ile13, Asn46, Tyr48, Asp54, Arg98, Gln133, Tyr137, Asn135, Asn138, and Asp140 were allowed to adopt different conformations during the induced-fit procedure. The Manα1,2Man and the Manα1,3Man dimannoside conformations were retrieved from the PDB database when in complex with the *P. angolensis* lectin (52); (Manα1,2Man: PDB ID 1Q8O Manα1,3Man: PDB ID: 1Q8P) (Table S2). The oligomannose-3 and oligomannose-6 conformations were taken from current work (Table S3). All other ligand structures were generated using the online carbohydrate builder of Glycam (53). For each ligand, ten docking poses that were energetically reasonable were kept while searching for the correct binding mode of the ligand. The decision to keep a trial pose was based on the computed energy for the interaction of the ligand with receptor of that pose. The ChemPLP fitness scoring function is the default in GOLD version 5.2 used to rank poses. Discovery Studio Visualizer 4.1 (Accelrys) was used for viewing. The figures of glycan structures were drawn using DrawGlycan-Symbol Nomenclature for Glycans (53).

### MD simulations and free energy calculations

MD trajectories were generated in the isothermal-isobaric ensemble at 300 K with the program NAMD2.12 using the CHARMM36 force field (54). Long-range electrostatic interactions were calculated using the particle-mesh Ewald method (55). A smoothing function was applied to truncate short-range electrostatic interactions. The Verlet-I/r-RESPA multiple time-step propagator was used to integrate the equation of motions using a time step of 2 and 4 fs for short- and long-range forces, respectively. All bonds involving hydrogen atoms were constrained using the Rattle algorithm. All systems (FimH–oligomannose complexes) were solvated and the ionic concentration was set to 0.15 M NaCl. All ionizable groups were assigned their standard protonation state as predicted by propKa (56). In total each molecular system comprised about 45,000 atoms. The equilibration was performed in three steps: (1) a 2.5-ns long equilibration of the solvent, being water and ions; (2) 2.5-ns long equilibration in which only the protein backbone was fixed, and (3) an unrestrained 2.5-ns long simulation were performed. This was followed by three independent 100-ns long MD production trajectories for each system.

Preexisting trajectories (from (17)) were prolonged to 100 ns each, for FimH–Man, FimH–Manα1,2Man, and FimH–Manα1,3Man dimannoside complexes (Table S2). For the oligomannosides, the best docking score poses were used to generate the trajectory (Table S3). Exceptions were the FimH complexes with Man3Gn2 (21), for Man3Gn2F1[6] and Man6Gn2 (this work), and trimannose (18), for which their respective crystal structures have been employed (Table S3). Furthermore, the stability of the divalent complex with Man6Gn2 was assessed using MD simulations using YASARA (57), version 21.8.27, with simulations of 100-ns duration.

As described (17), the free energy of binding, ΔG, was computed for each of the different glycans using a hybrid MM-PBSA approach as implemented in g_mmpbsa (58). The total of 3000 frames, extracted every 0.1 ns from the three independent simulations, was used for the calculations. The values and standard errors shown in Tables S2 and S3 represent the mean of all three simulations. To account for the larger glycans, the selection of protein residues was enlarged in than the previous study and encloses the binding region formed by the residues 1 to 4, 10 to 17, 44 to 56, 94 to 102, and 133 to 143 (Fig. S7).

### Detection of direct binding to FimH in the LEctPROFILE kit assay

BSA-dimmanose conjugates (DEXTRA Laboratories) were biotinylated using the EZ-Link NHS-LC-Biotin labeling kit (Thermo Fisher Scientific). The FimH LEctPROFILE kit is designed in a 96-well format (15, 16). Fifty μl of each sample at three different concentrations and preliminary labeled with biotin were added in each well of the plate. Each of the three concentrations has been repeated six times. Upon 1 h incubation at room temperature, the 96-well microplate was washed three times with 200 μl of PBS Tween 0.05%. Then, 50 mL of the streptavidin-DTAF (DichloroTriazinylAminoFluorescein) was added in each well of the plate and the plate was kept 30 min protected from light. The plate was washed again three times with 200 μl of PBS Tween 0.05% and finally, 100 μl of PBS was added for the read-out (λ_excitation_ = 490 nm, λ_emission_ = 520 nm).

### SPR measurements of FimH-BSA-dimannose and dual mixes

SPR detection was used for the study of the kinetics of association and dissociation of FimH with Manα1,3Man-BSA, Manα1,2Man-BSA, Manα1,4Man-BSA, Manα1,6Man-BSA, and dual mixes presenting dimannosides found on high-mannose N-glycans. The measurements were made in a single cycle kinetics, regeneration-free, mode using a BiacoreT200 (Cytiva). The single dimannoside-BSA or equimolar mixes were immobilized *via* the primary amino groups on BSA, at low rates in a single flow cell on a CM5 sensor chip, through EDC/NHS activation. The reference flow cell was left untreated. The binding interactions were studied at different concentrations of FimH (20 μM, 4 μM, 0.8 μM, 0.16 μM, 0.032 μM) at a flow rate of 30 μl/min in 20 mM Hepes pH 7.4, 150 mM NaCl and 0,005% Tween and at 298 K. Full regeneration of the sensor chip was not possible and a new sensor chip was prepared for at least one repetition. The data analysis was performed using the BIAevaluation software.

### SPR measurements of FimH binding with Man3Gn2 and Man3Gn2F1[6]

To obtain the affinity between FimH and the glycan structures Man3Gn2 or Man3Gn2F1[6], SPR measurements were used on a Biacore3000 (Cytiva). The sugar Man3Gn2 and Man3Gn2F1[6] were immobilised respectively in flow cells 2 and 4 on a CM5 sensor chip. Flow cells 1 and 3 were left untreated and served as a reference. The binding interactions were studied at a 2-fold dilution series of FimH (8.58 μM, 4.29 μM, 2.15 μM, 1.07 μM, 0.57 μM; 0.26 μM; 0.13 μM), in the running buffer containing 20 mM Hepes, 150 mM NaCl, and 0.005% Tween at a flow rate of 30 μl min^−1^ and at 298 K. Regeneration performed using 50 mM NaOH allowed repeats on the same sensor chip. The data analysis was performed using the BIAevaluation software.

### SPR measurements of FimH binding to ω1-glycoproteins carrying Man3Gn2 modified with xylose and fucose

To obtain the affinity between FimH and ω1-glycoproteins, the single-cycle kinetics SPR technique was applied on a BIAcoreT200 (Cytiva). The ω1-glycoproteins had been produced in glycan-engineered plants and purified as described (59). The ω1-glycoproteins were immobilized in a single flow cell on a CM5 sensor chip, *via* primary amino groups using EDC/NHS activation. Flow cell, one of the CM5 sensor chip, was left untreated and served as a reference. The binding interactions were studied at different FimH concentrations (14.4 μM, 7.17 μM, 3.60 μM, 1.80 μM, 0.9 μM), in the running buffer with 20 mM Hepes, 150 mM NaCl and 0.005% Tween, at a flow rate of 30 μl min^−1^ and at 298 K. A new sensor chip was required for each repetition. The data analysis was performed using the BIAevaluation software.

## Data availability

PDB https://doi.org/10.2210/pdb7BHD/pdb: FimH in complex with alpha1,6 core-fucosylated oligomannose-3, crystallized in the trigonal space group, and entry https://doi.org/10.2210/pdb8BXY/pdb (STARANISO data processing).

PDB https://doi.org/10.2210/pdb7QUO/pdb: FimH lectin domain in complex with oligomannose-6, and entry https://doi.org/10.2210/pdb8BY3/pdb (STARANISO data processing).

All other data are available in the article, other raw data files are available through the authors.

## Supporting information

This article contains supporting information (65, 66).

## Conflict of interest

N.-C. R. is CEO and shareholder of Asparia Glycomics S.L., a company commercializing glycoscience products and services.
