# Supplementary material for: Structural insights into a cooperative switch between one and two FimH bacterial adhesins binding pauci- and high-mannose type N-glycan receptors
Source: J Biol Chem. 2023 Mar 20;299(5):104627. doi: 10.1016/j.jbc.2023.104627 (PMC10127133; doi:10.1016/j.jbc.2023.104627)
Supplement: Supporting Figures S1–S10 and Tables S1–S4 [file mmc1.docx]

**Structural insights into a cooperative switch between one and two FimH bacterial adhesins binding pauci- and high-mannose type *N*-glycan receptors**

Eva-Maria Krammer*^1#^*, Clarisse Bridot*^1#^*, Sonia Serna*^2#^*, Begoña Echeverria*^2^*, Shubham Semwal*^1^,* Benoît Roubinet*^3^*, Kim van Noort^4^, Ruud H. P Wilbers^4^, Gleb Bourenkov^5^, Jérôme de Ruyck*^1^*, Ludovic Landemarre*^3^*, Niels Reichardt*^2,6^*, Julie Bouckaert*^1*^*

*^1^Unité de Glycobiologie Structurale et Fonctionnelle (UGSF), UMR8576 du CNRS and the University of Lille, 50 Avenue Halley, 59658 Villeneuve d'Ascq, France;*

*^2^Glycotechnology Group, Basque Research and Technology Alliance (BRTA), CIC biomaGUNE, Paseo Miramon 194, 20014 Donostia, Spain*

*^3^GLYcoDiag, 2 Rue du Cristal, 45100 Orléans, France;*

*^4^Laboratory of Nematology, Plant Science Group, Wageningen University and Research, Droevendaalsesteeg 1, 6708 Wageningen, The Netherlands;*

*^5^European Molecular Biology Laboratory (EMBL), Hamburg Unit c/o DESY, Notkestrasse 85, 22607 Hamburg, Germany.*

*^6^CIBER-BBN, Paseo Miramon 194, 20014 Donostia, Spain*

# contributed equally

* corresponding author: julie.bouckaert@univ-lille.fr

*Running title:* *The ABC of oligomannose-6 binding to the FimH adhesin*

*Keywords* FimH, *N*‐glycan, multivalency, paucimannose, oligomannose-3, core fucose, oligomannose-6, kinetics, crystal structure, bacterial adhesion, cooperativity

**Index**

Glycan microarray: composition, summary of results and heat map 3

Monovalent binding of the Man3Gn2F1[6] *N*-glycan to FimH in the crystal structure 7

FimH binding to ω1-glycoproteins using SPR kinetics 8

Serial crystallography using on-salt-crystal FimH protein – Man6Gn2 crystals 10

FimH – Man3Gn2F1(6) and FimH – Man6Gn2 crystal structure data 12

Mannoside- FimH complexes in molecular dynamics simulations 14

Protein-carbohydrate interactions in the crystal structures 16

MALDI-TOF spectrum of synthesized Man3Gn2F1[6] 19

^1^H-NMR spectrum in D2O spectrum of synthesized Man3Gn2F1[6] 20

References 20

Glycan microarray: composition, summary of results and heat map


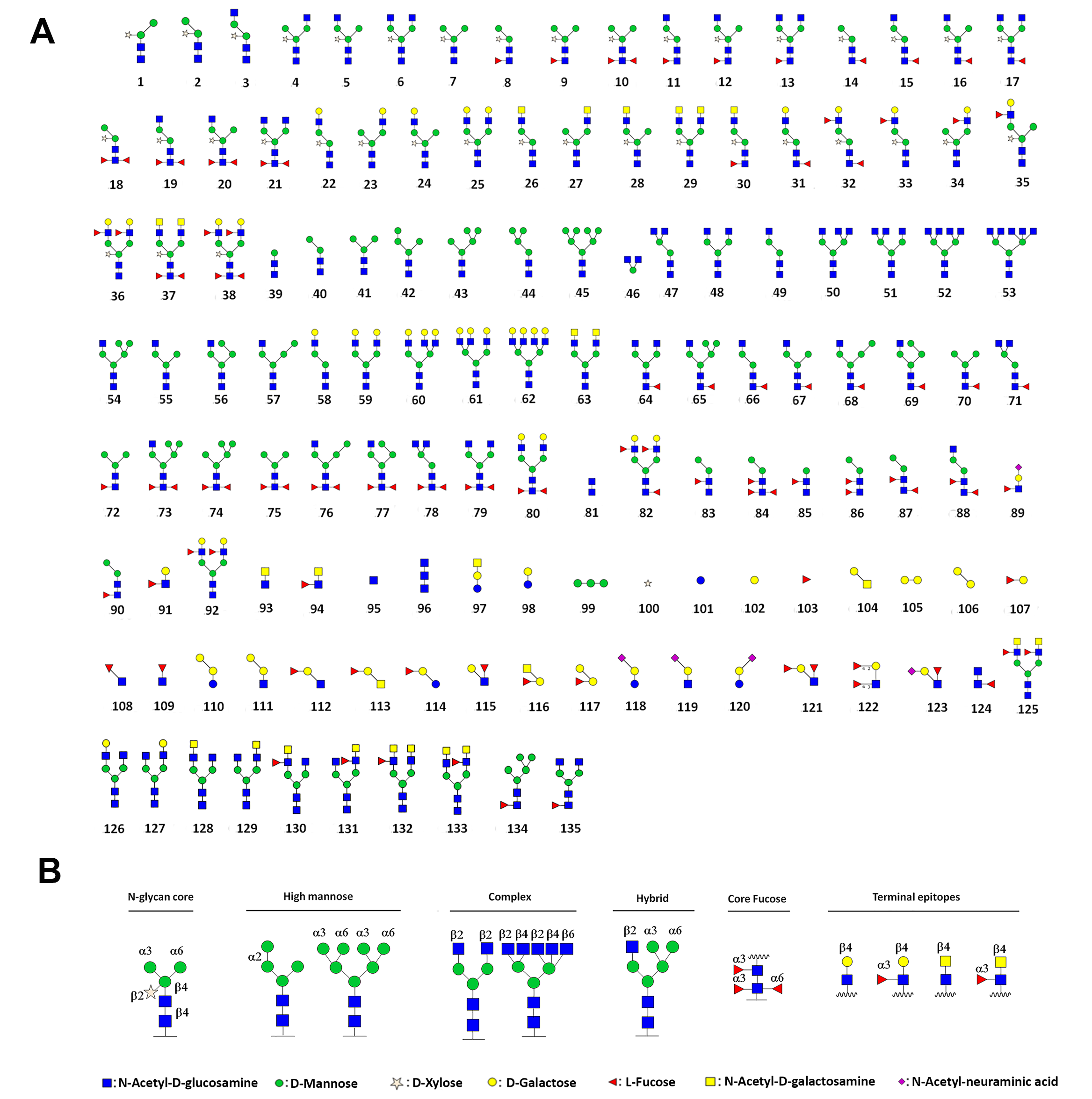


**Figure S1**: Glycan array: **A**. Pictogram representation of glycan structures printed on glycan microarrays. **B**. Specification of glycosidic bond configurations.

Monosaccharide symbol nomenclature according to Consortium of Functional Glycomics recommendations http://www.functionalglycomics.org/static/consortium/Nomenclature.shtml

**
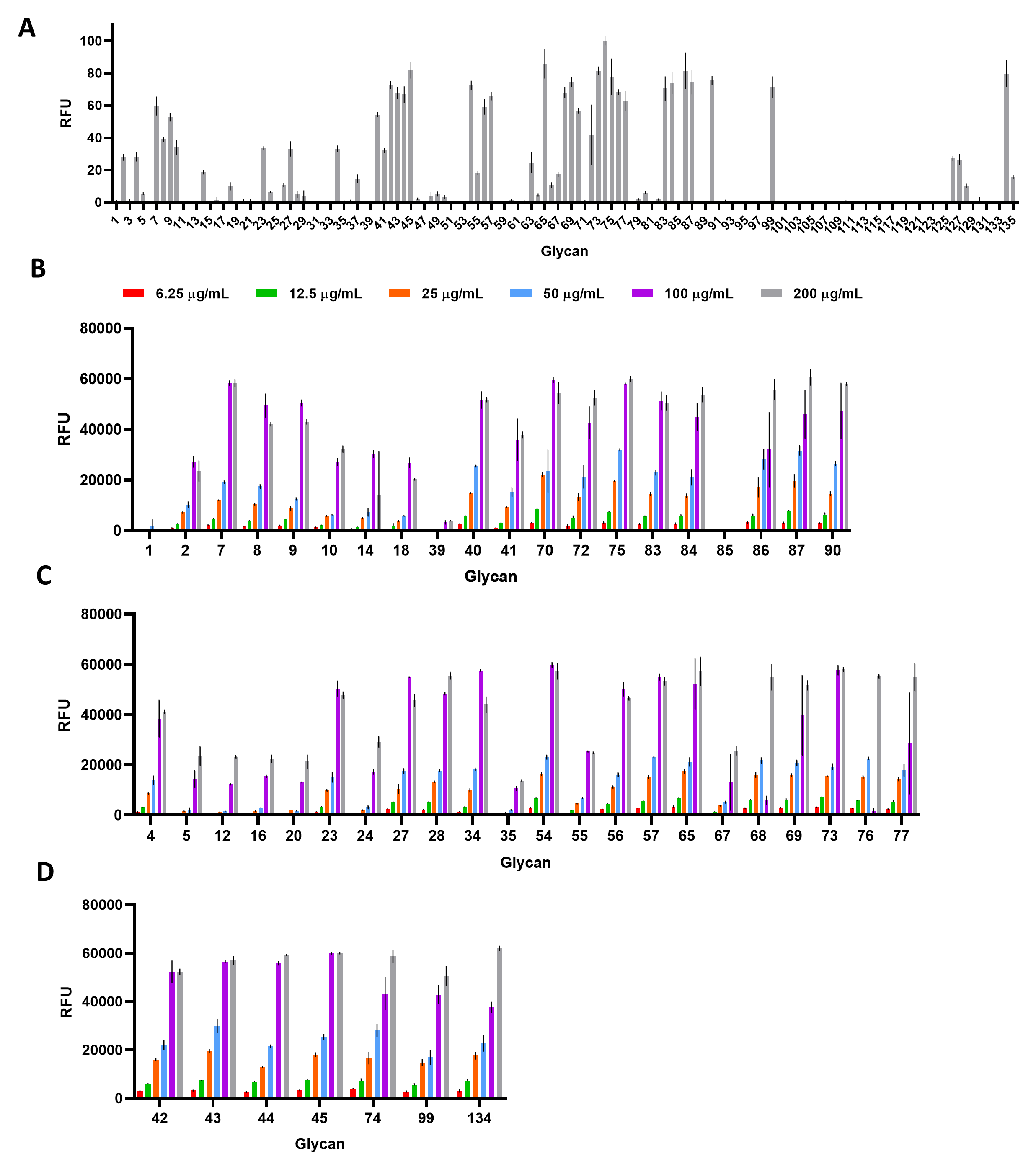
Figure S2**. FimH binding profile on glycan microarrays. FimH was incubated at different concentrations: 6.25, 12.5, 25, 50, 100 and 200 μg/mL/ and detection of binding was realized using polyclonal rabbit anti-FimH followed by anti-rabbit-IgG-Alexa555. **A.** Here shown is the binding profile of FimH at 25 μg/mL/anti FimH/anti rabbit-IgG-555. Each histogram represents the mean normalized RFU values for four spots, with error bars indicating the standard error. **B**, **C** and **D**: The dose-dependent response of six FimH concentrations on paucimannose (**B**), hybrid (**C**), and high-mannose (**D**) glycan structures.

**Figure S3**: Dual colour heat map representation of FimH, ConA and GNA interactions with selected structures (high mannose, hybrid and paucimannose *N*-glycan type structures) printed on glycan microarray. RFU values were previously normalized to maximum RFU for each lectin. Hierarchical clustering is based on Euclidean distance (average linkage method). Red = high binding. Green = low to no binding.

# Monovalent binding of the Man3Gn2F1[6] *N*-glycan to FimH in the crystal structure

**
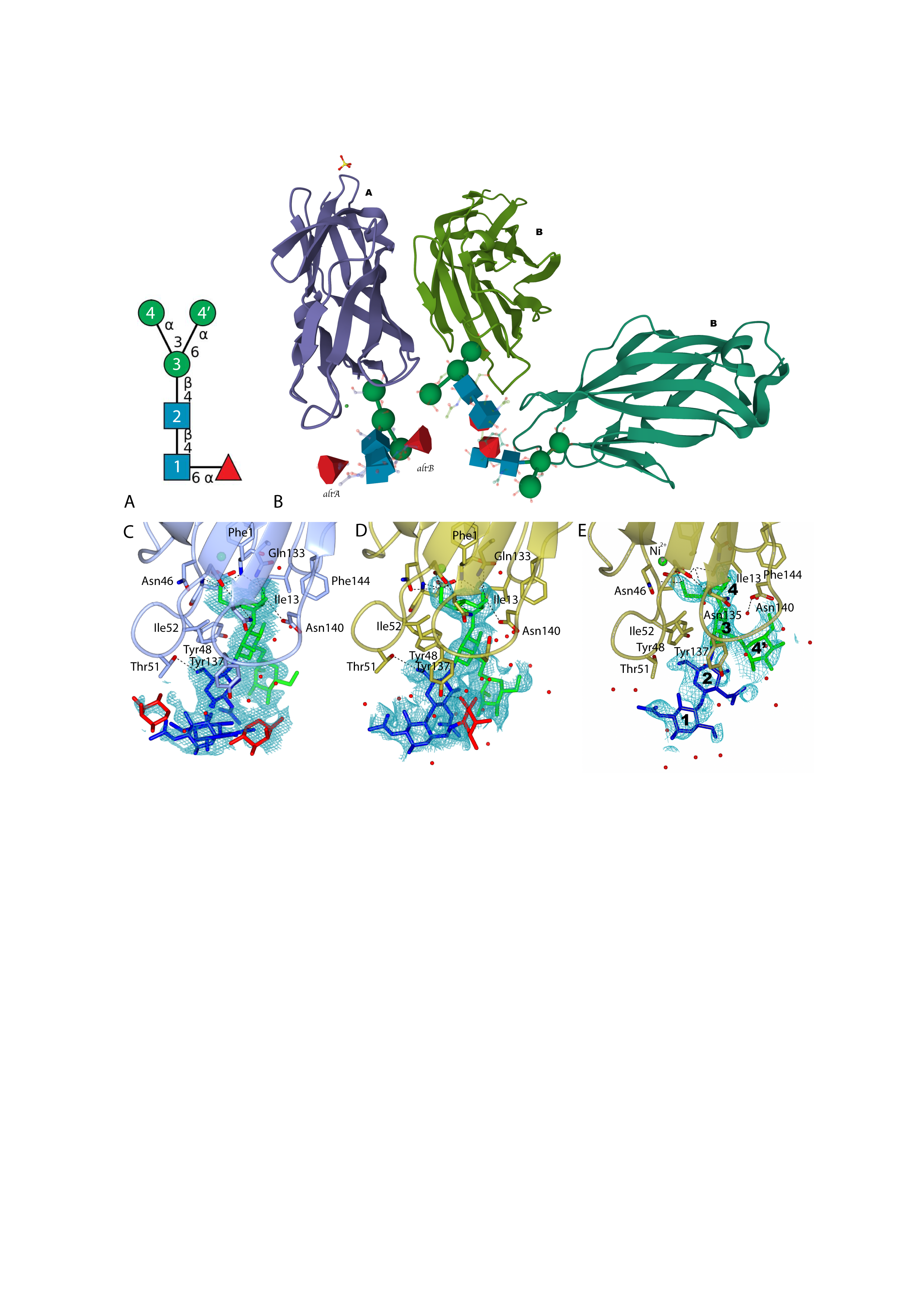
Figure S4**: Monovalent binding of the Man3Gn2F1[6] (*N*-glycan # 70 in **Figure S1**) ligand with FimH, in the crystal structure (PDB entry 7BHD). **A**. SNFG (1) presentation of Man3Gn2F1[6] using DrawGlycan-SNFG (2). **B**. Crystal packing of a trio of FimH complexes showing the two alternate conformations of the α1,6 core fucosylated chitobiose of the ligand (chain C) bound to FimH (chain A), and the close contact of the two fucose residues (chain D) between two symmetry-related FimH protein chains B, using Mol* (3). **C**, **D**, **E**. Omit electron densities at 0.7 σ of Man3Gn2F1[6] in FimH chains A (**C**) and B (**D**) in the crystal structure with PDB entry 7BHD, and of Man3Gn2 in FimH chain B of the crystal structure with PDB entry 2VCO (4). These figures have been designed using CCP4mg (5,6), with the omit density of the *N*-glycan ligand calculated using Privateer (7).

# FimH binding to ω1-glycoproteins using SPR kinetics


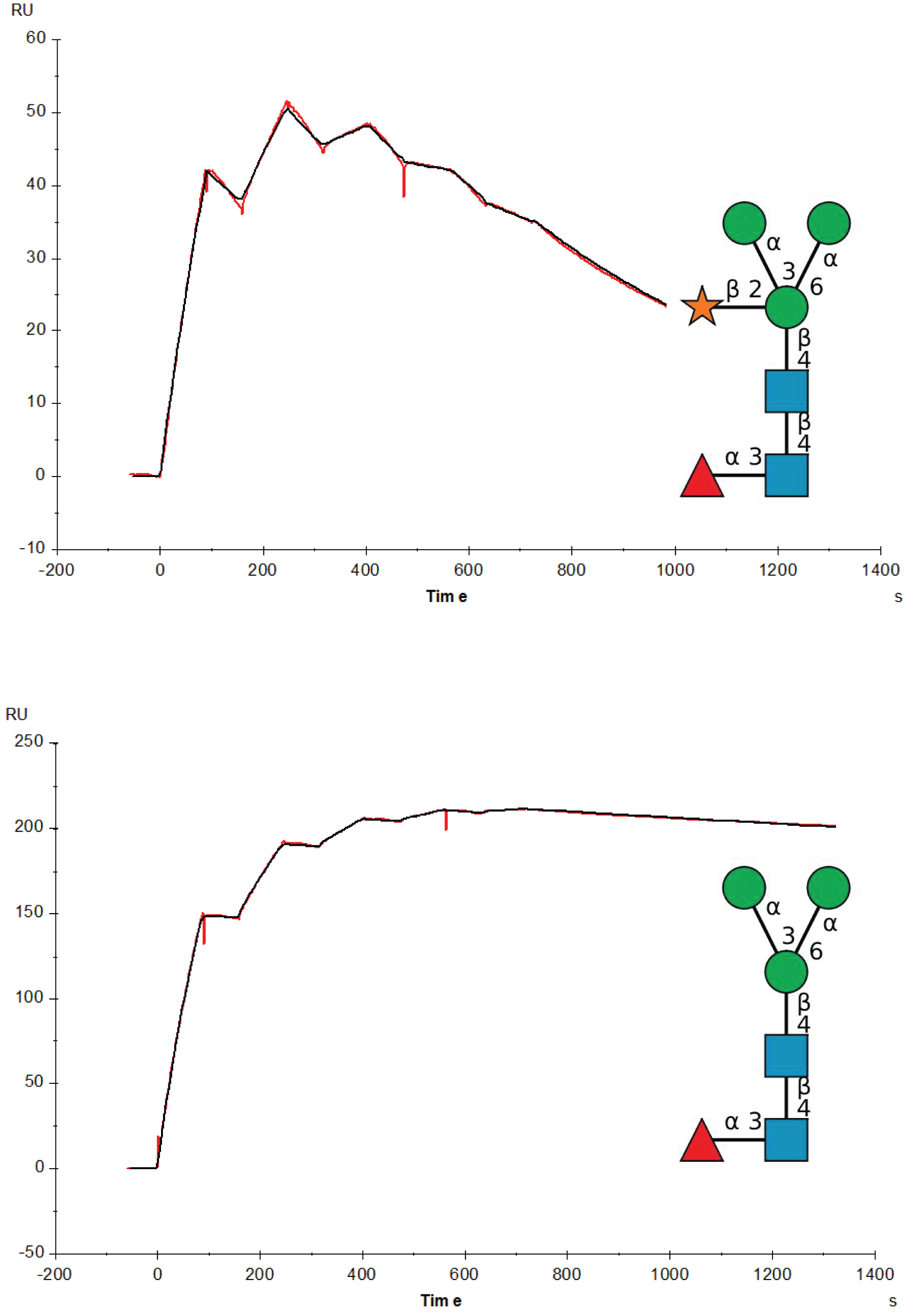


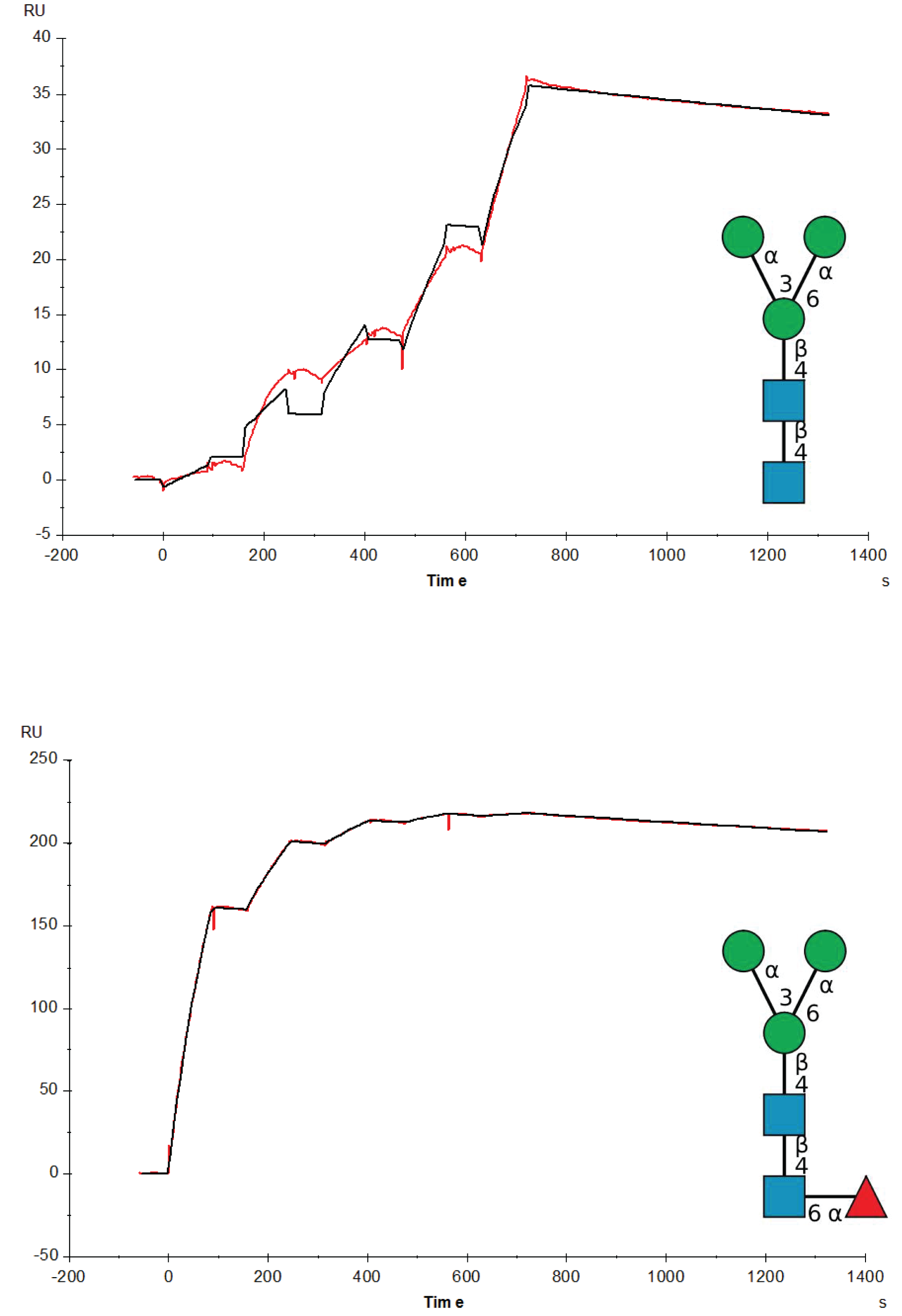


**Figure S5**: SPR sensorgrams to study the kinetics of binding of paucimannose carrying ω1-glycoproteins (measured (black lines) and fitted (red lines) values in **Table 1**), with the predominant glycan structure shown in the inset.

# Serial crystallography using on-salt-crystal FimH protein – Man6Gn2 crystals


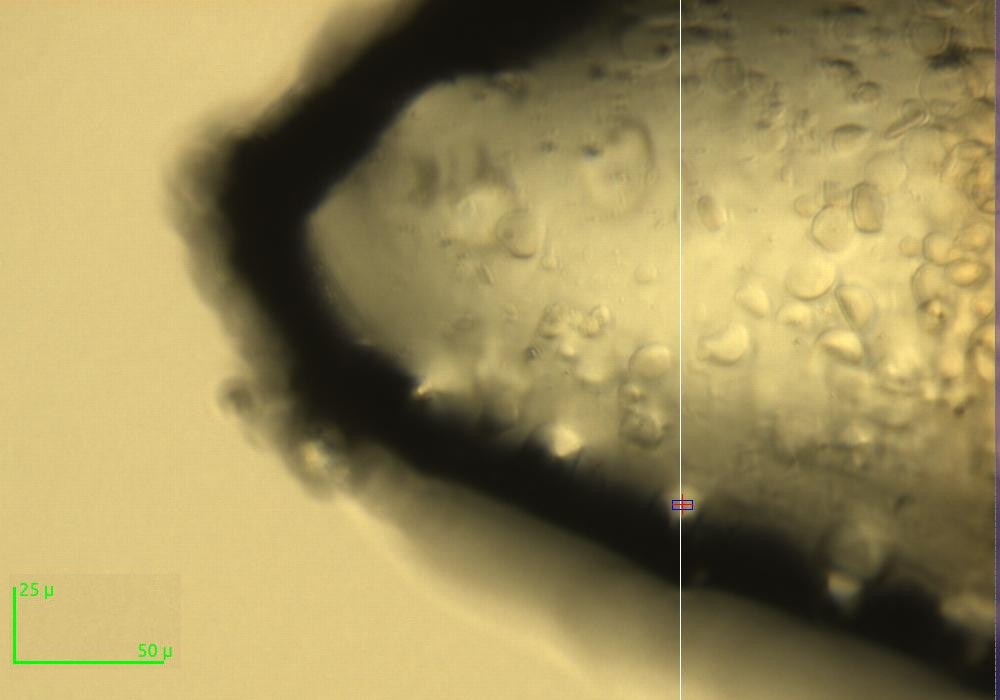


(**A**)


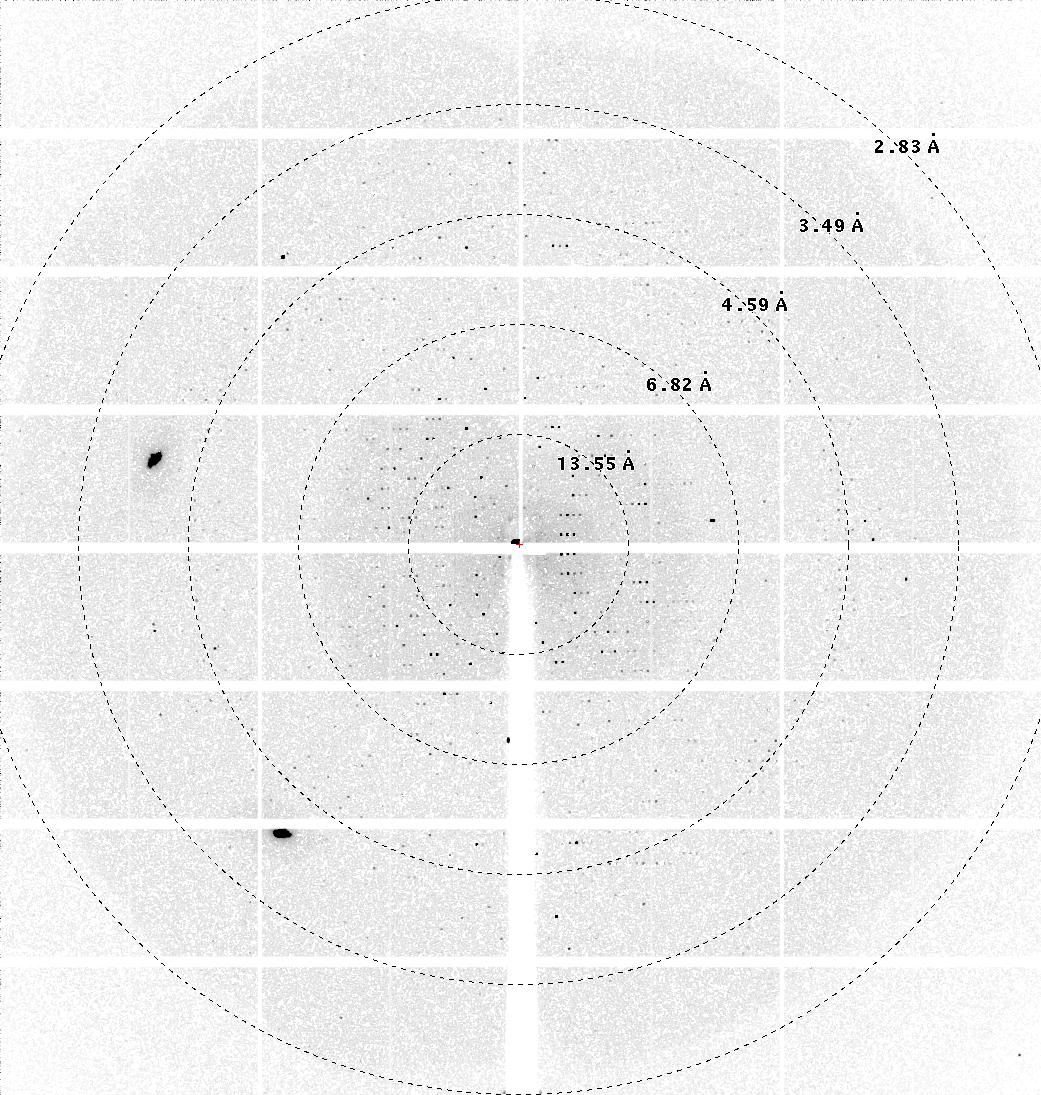


(**B**)

**Figure S6**: **A**. Selection of a FimH-oligomannose-6 crystal grown on top of a lithium sulphate salt crystal. **A** is an image of sample mount; red cross and blue rectangle indicate the X-ray beam positioned on the FimH - oligomannose-6 crystal from which the diffraction pattern in **B** was measured. **B** is the diffraction pattern obtained by summation of 20 x 0.1º rotation (2º total rotation). One can see strong diffuse reflections from the salt "substrate", as well the X-ray diffraction pattern from the FimH-oligomannose-6 crystal.

# FimH – Man3Gn2F1(6) and FimH – Man6Gn2 crystal structure data

**Table S1.1**. Data collection and refinement statistics.

|  | **FimH - Man3Gn2F1[6]** | **FimH – Man6Gn2** |
| --- | --- | --- |
| **PDB entry code** | 7BHD | 7QUO |
| **Resolution range^*^** | 78.99 – 1.40 (1.48 - 1.40) | 86.97 – 3.00 (3.08 - 3.00) |
| **Space group** | P 3_1_ 2 1 | P 6_1_ 2 2 |
| **Unit cell constants**  **a, b, c (Å)**  **α, β, γ** **(°)** | 91.21, 91.21, 79.66  90.00, 90.00, 120.00 | 153.11, 153.11, 230.41,  90.00, 90.00, 120.00 |
| **Number of molecules in the asymmetric unit** | 2 | 4 |
| **Total reflections** | 1445001 (184769) | 355380 (25825) |
| **Unique reflections** | 75457 (11867) | 32556 (2344) |
| **Multiplicity** | 19.15 (15.57) | 5.93 (5.82) |
| **Completeness (%)** | 99.5 (97.9) | 99.0 (99.7) |
| **< I/sigma(I) >** | 17.01 (0.82) | 3.13 (0.40) |
| **Wilson B-factor (Å^2^)** | 24.39 | 29.96 |
| **R-merge** | 0.075 (4.192) | 0.68 (4.97) |
| **R-meas.** | 0.077 (4.331) | 0.67 (5.25) |
| **CC1/2** | 1.0 (0.2) | 0.90 (0.06) |
| **Reflections used in refinement** | 69952 (8199) | 32551 (3177) |
| **Reflections used for R-free** | 1853 (226) | 1992 (195) |
| **R-work** | 0.1708 (0.3632) | 0.2358 (0.3317) |
| **R-free** | 0.1979 (0.4206) | 0.2756 (0.3619) |
| **Coordinate error (Å) (maximum-likelihood based)** | 0.22 | 0.45 |
| **Phase error (°) (maximum-likelihood based)** | 26.06 | 25.03 |
| **Clash score** | 7.74 | 5.40 |
|  |  |  |
| **Number of non-hydrogen atoms** | 3053 | 5154 |
| **macromolecules** | 2404 | 4784 |
| **ligands** | 236 | 368 |
| **solvent** | 483 | 166 |
| **Protein residues** | 316 | 624 |
| **RMS (bonds)** | 0.024 | 0.003 |
| **RMS (angles)** | 2.06 | 0.815 |
| **Ramachandran favoured (%)** | 97.41 | 97.44 |
| **Ramachandran allowed (%)** | 2.59 | 2.40 |
| **Ramachandran outliers (%)** | 0.00 | 0.16 |
| **Rotamer outliers (%)** | 0.37 | 0.56 |
|  |  |  |
| **Average B-factor (Å^2^)** | 34.84 | 44.55 |
| **macromolecules** | 31.37 | 40.09 |
| **ligands** | 59.52 | 67.46 |
| **solvent** | 43.60 | 20.66 |

**^*^** Statistics for the highest-resolution shell are shown between parentheses.

|  | **FimH - Man3Gn2F1[6]** | **FimH – Man6Gn2** |
| --- | --- | --- |
| **PDB entry code** | 8BXY | 8BY3 |
| **Resolution range^*^** | 56.08 – 1.45 (1.56 - 1.45) | 114.92 – 3.19 (3.34 - 3.19) |
| **Space group** | P 3_1_ 2 1 | P 6_1_ 2 2 |
| **Unit cell constants**  **a, b, c (Å)**  **α, β, γ** **(°)** | 91.06, 91.06, 79.58  90.00, 90.00, 120.00 | 153.11, 153.11, 230.41,  90.00, 90.00, 120.00 |
| **Number of molecules in the asymmetric unit** | 2 | 4 |
| **Total reflections** | 1130391 (47660) | 267927 (15799) |
| **Unique reflections** | 56199 (2811) | 24192 (1209) |
| **Multiplicity** | 20.1 (17.0) | 11.1 (13.1) |
| **Completeness (%)** | 82.9 (21.9) | 88.5 (34.1) |
| **< I/sigma(I) >** | 19.3 (1.6) | 5.0 (1.6) |
| **Wilson B-factor (Å^2^)** | 24.4 | 69.9 |
| **R-merge** | 0.075 (1.903) | 0.613 (1.767) |
| **R-meas.** | 0.077 (1.961) | 0.643 (1.838) |
| **R-pim** | 0.017 (0.462) | 0.188 (0.502) |
| **CC1/2** | 1.000 (0.615) | 0.95 (0.58) |
|  |  |  |
| **Number of reflections used in refinement** | 54717 (351) | 23169 (489) |
| **Reflections used for R-free** | 1481 (14) | 1532 (27) |
| **R-work** | 0.153 (0.300) | 0.204 (0.271) |
| **R-free** | 0.205 (0.327) | 0.238 (0.282) |
| **Coordinate error (Å) based on maximum likelihood** | 0.059 | 0.295 |
| **Clash score** | 3.19 | 8.26 |
|  |  |  |
| **Number of non-hydrogen atoms (<B_fact_> (Å^2^))** | 2900 (32.7) | 5153 (67.0) |
| **protein** | 2404 (29.2) | 4784 (67.9) |
| ***N*-glycan** | 149 (70.9) | 200 (88.7) |
| **ions** | 2 (35.7) | 4 (71.0) |
| **waters** | 345 (40.4) | 165 (46.7) |
| **Protein residues** | 316 | 624 |
| **RMS (bonds) Å** | 0.016 | 0.018 |
| **RMS (angles) º** | 1.88 | 1.77 |
| **B values error (Å^2^) based on maximum likelihood** | 3.674 | 17.795 |
|  |  |  |
| **Ramachandran favoured (%)** | 97.09 | 97.28 |
| **Ramachandran allowed (%)** | 2.59 | 2.72 |
| **Ramachandran outliers (%)** | 0.32 | 0.00 |
| **Rotamer outliers (%)** | 1.12 | 1.88 |

**Table S1.2**. Data statistics upon anisotropic re-scaling and merging and re-refinement statistics.

**^*^**Statistics for the highest-resolution shell are shown in parentheses.

#
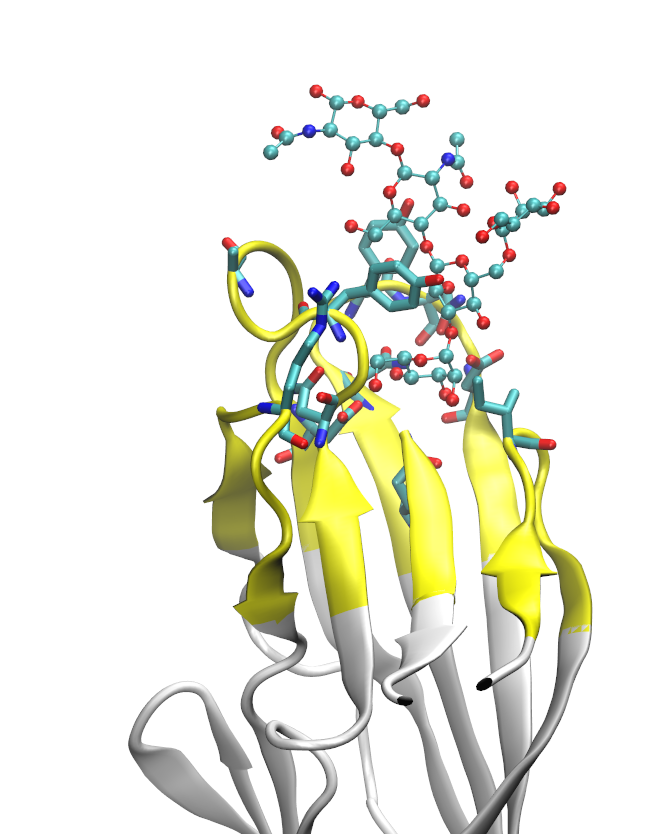
Mannoside- FimH complexes in molecular dynamics simulations

**Figure S7**: Region (yellow) on the FimH lectin domain for the calculation of the binding energetics of the larger glycans from MD simulations (Tables S3 and S4).

**Table S2**: Energetics of binding of the different mannosides calculated from the MD simulations.

| Compound | Energy contribution (kcal.mol^-1^) | | | | | | |
| --- | --- | --- | --- | --- | --- | --- | --- |
|  | ΔE_ele_ | ΔE_vdw_ | ΔE_int_ | ΔG_solv POLAR_ | ΔG_solv UNPOLAR_ | ΔG_solv_ | ΔG_binding_ |
| Man | -287.4±0.5 | -37.1±0.3 | -324.5±0.5 | 120.5±0.2 | -9.2±0.0 | 111.3±0.2 | -213.2±0.4 |
| Manα1,2Man | -292.2±0.5 | -60.2±0.3 | -352.4±0.5 | 144.4±0.4 | -12.4±0.0 | 131.5±0.4 | -220.5±0.8 |
| Manα1,3Man | -315.6±0.8 | -60.9±0.3 | -376.5±0.8 | 163.8±0.5 | -12.2±0.0 | 151.4±0.5 | -224.9±0.5 |
| Manα1,4Man | -290.0±0.6 | -59.4±0.3 | -349.4±0.6 | 143.1±0.4 | -11.9±0.0 | 132.8±0.4 | -218.8±0.4 |
| Manα1,6Man | -302.5±0.8 | -55.6±0.3 | -358.1±0.7 | 155.1±0.4 | -12.0±0.0 | 143.1±0.4 | -215.0±0.5 |

**Table S3** Computation of binding energies, of different oligomannoses of growing length, based on molecular dynamics simulations (3 repetitions, each trajectory 100 ns long) using a hybrid MM-PBSA approach (8). The complexes of the FimH lectin domain with a glycan structure (labelled, where applicable, with the glycan’s number on the glycan array) originate from crystal structures (labelled with PDB code), or are derived from docking, eventually alternatingly a non-reducing-end mannose of each different oligomannose arm, into the mannose monosaccharide-binding pocket of the FimH lectin.

| Compound  *Glycan array n°* | Docking | Binding to the dimannose ending | Energy contribution  (kcal.mol^-1^) | | | | | | |
| --- | --- | --- | --- | --- | --- | --- | --- | --- | --- |
|  | PDB code |  |  |  |  |  |  |  |  |
|  |  |  | ΔE_ele_ | ΔE_vdw_ | ΔE_int_ | ΔG_solv POLAR_ | ΔG_solv UNPOLAR_ | ΔG_solv_ | ΔG_binding_ |
| 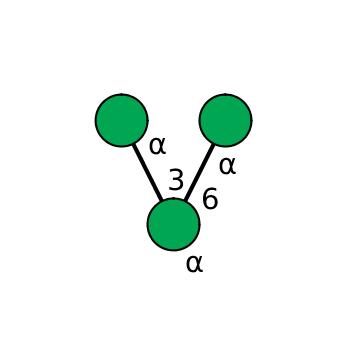 | 6GTW | Manα1,3Man | -292.9±0.5 | -64.2±0.3 | -356.1±0.5 | 144.0±0.4 | -12.7±0.0 | 131.3±0.4 | -224.8±0.4 |
|  | 6GTW | Manα1,6Man | -295.4±0.6 | -62.9±0.6 | -358.3±0.6 | 149.8±0.7 | -13.2±0.0 | 136.6±0.3 | -221.7±0.5 |
| *41*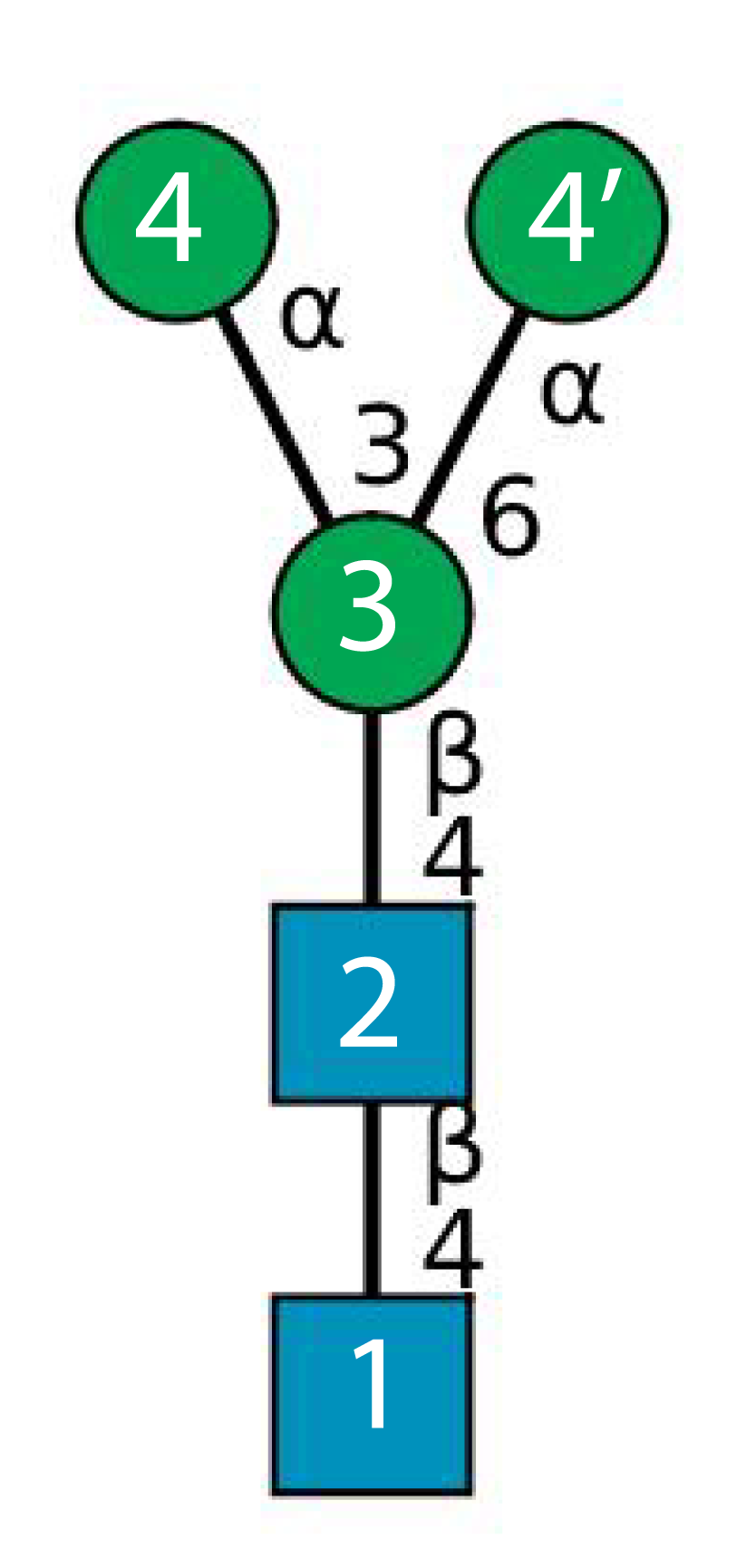 | 2VCO | Manα1,3Man | -335.6±0.9 | -96.4±0.3 | -432.0±0.9 | 206.5±0.7 | -17.3±0.0 | 189.2±0.6 | -242.8±0.6 |
|  | 2VCO | Manα1,6Man | -304.2±0.7 | -84.8±0.3 | -389.0±0.7 | 169.9±0.5 | -16.1±0.0 | 153.8±0.5 | -235.2±0.5 |
| *40*_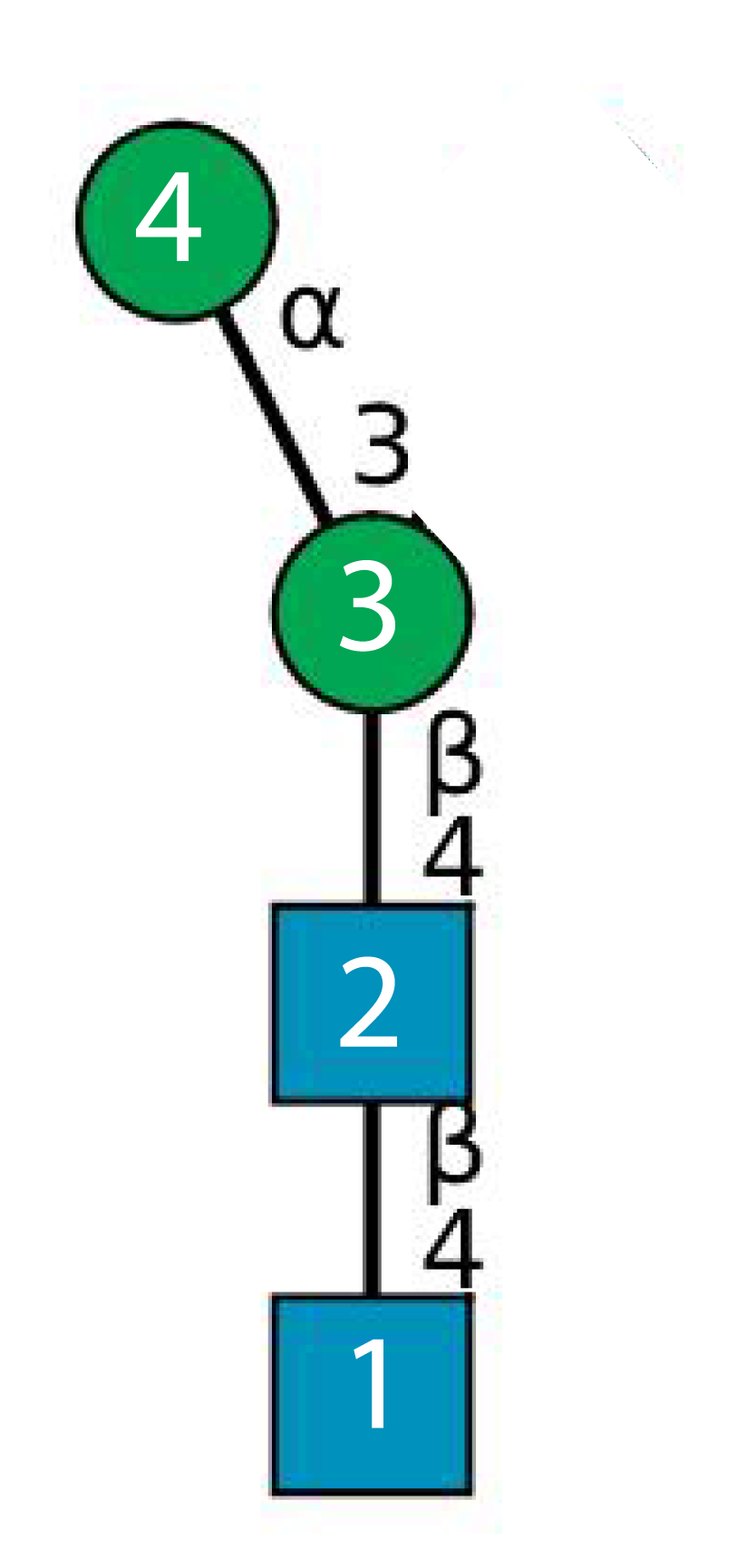_ | Docking | Manα1,3Man | -307.8±0.6 | -86.8±0.3 | -394.6±0.6 | 174.2±0.4 | -16.0±0.0 | 158.2±0.4 | -236.4±0.5 |
| 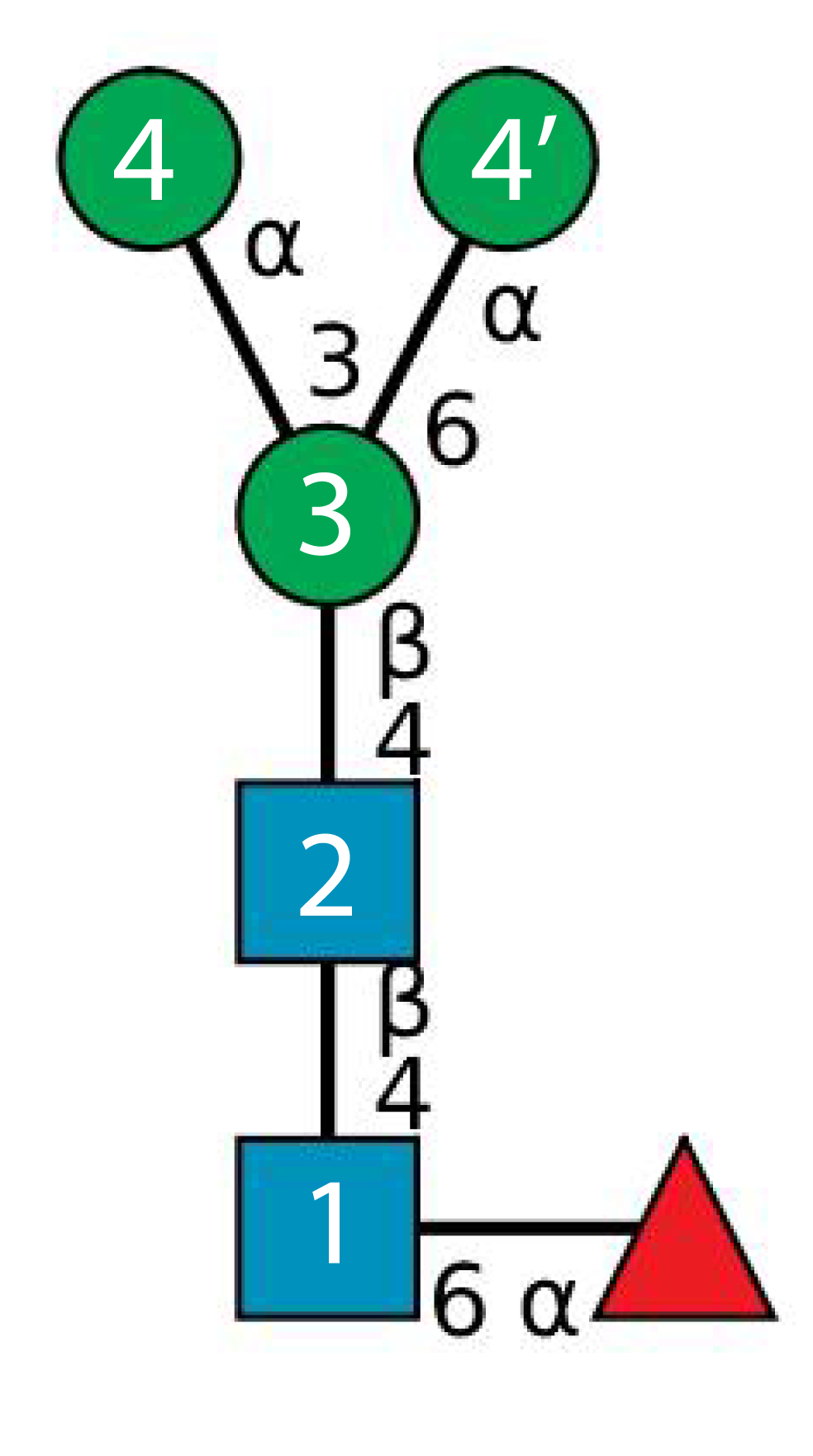  *70* | Docking | Manα1,3Man | -329.1±0.9 | -97.9±0.4 | -427.0±0.9 | 213.1±0.7 | -17.2±0.0 | 195.9±0.7 | -231.1±0.7 |
|  | Docking | Manα1,6Man | -335.5±1.3 | -85.6±0.4 | -421.2±1.4 | 210.1±1.0 | -16.5±0.0 | 193.9±0.9 | -227.6±0.9 |
|  | altA < 7BHD A | Manα1,3Man | -315.6±0.7 | -96.9±0.3 | -412.6±0.8 | 201.9±0.6 | -17.2±0.0 | 184.8±0.6 | -227.8±0.6 |
|  | altB < 7BHD A | Manα1,3Man | -322.8±0.6 | -97.6±0.4 | -420.4±0.7 | 204.2±0.5 | -17.0±0.4 | 187.2±0.5 | -233.2±0.6 |
|  | 7BHD B | Manα1,3Man | -311.2±0.5 | -97.0±0.3 | -408.2±0.5 | 192.0±0.4 | -16.6±0.0 | 175.4±0.4 | -232.9±0.5 |
| 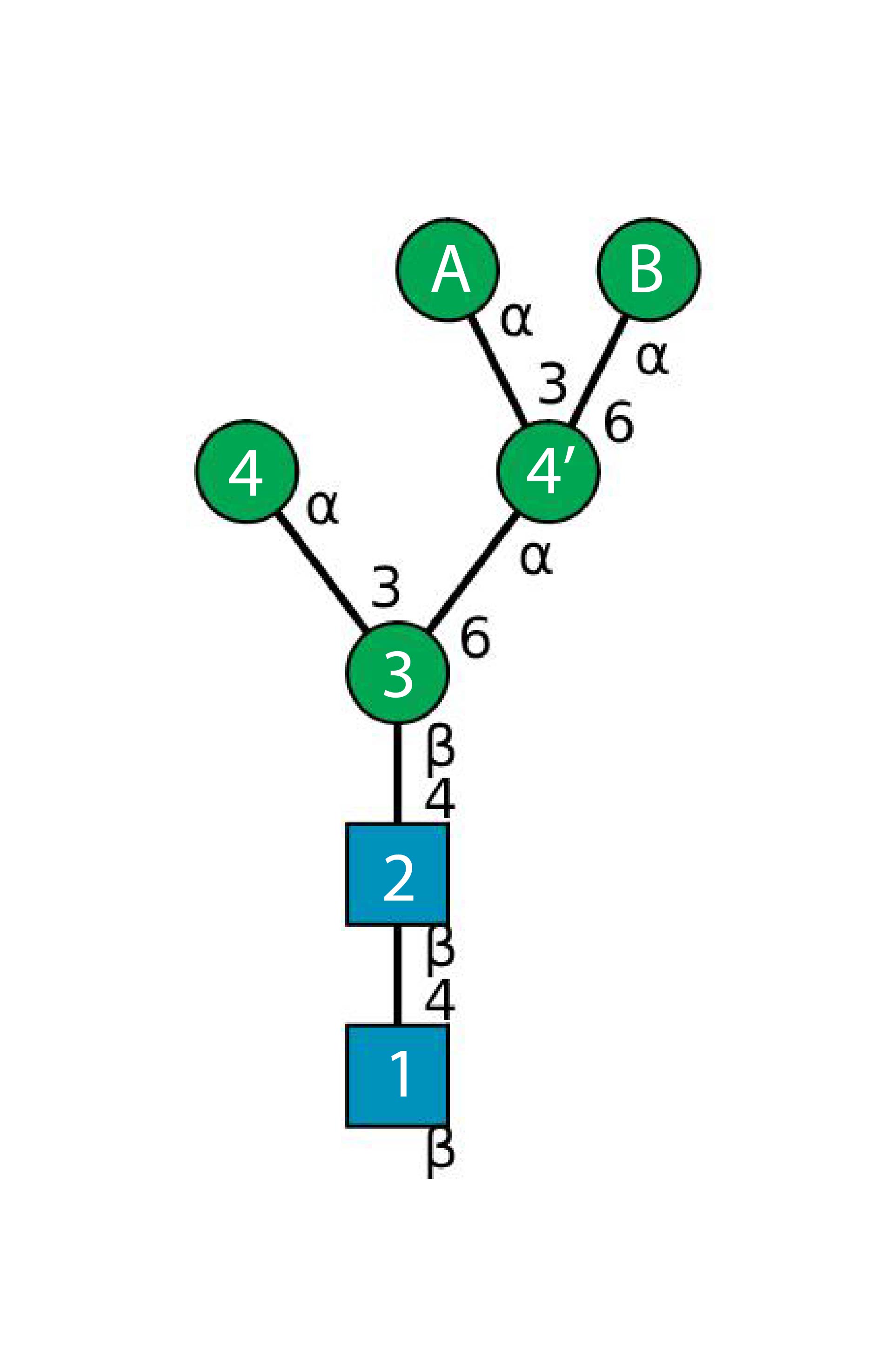*43* | Docking | Manα1,3Man (4) | -382.4±1.2 | -115.3±0.4 | -497.7±1.3 | 270.4±1.1 | -21.1±0.0 | 249.3±1.1 | -248.4±0.7 |
|  | Docking | Manα1,3Man(A) | -337.8±1.2 | -114.6±0.5 | -472.4±1.4 | 242.9±1.1 | -20.6±0.0 | 222.3±1.1 | -250.1±0.8 |
|  | Docking | Manα1,6Man (B) | -312.8±0.8 | -98.9±0.4 | -411.6±1.0 | 204.0±0.7 | -17.5±0.0 | 186.5±0.3 | -225.2±0.8 |
| _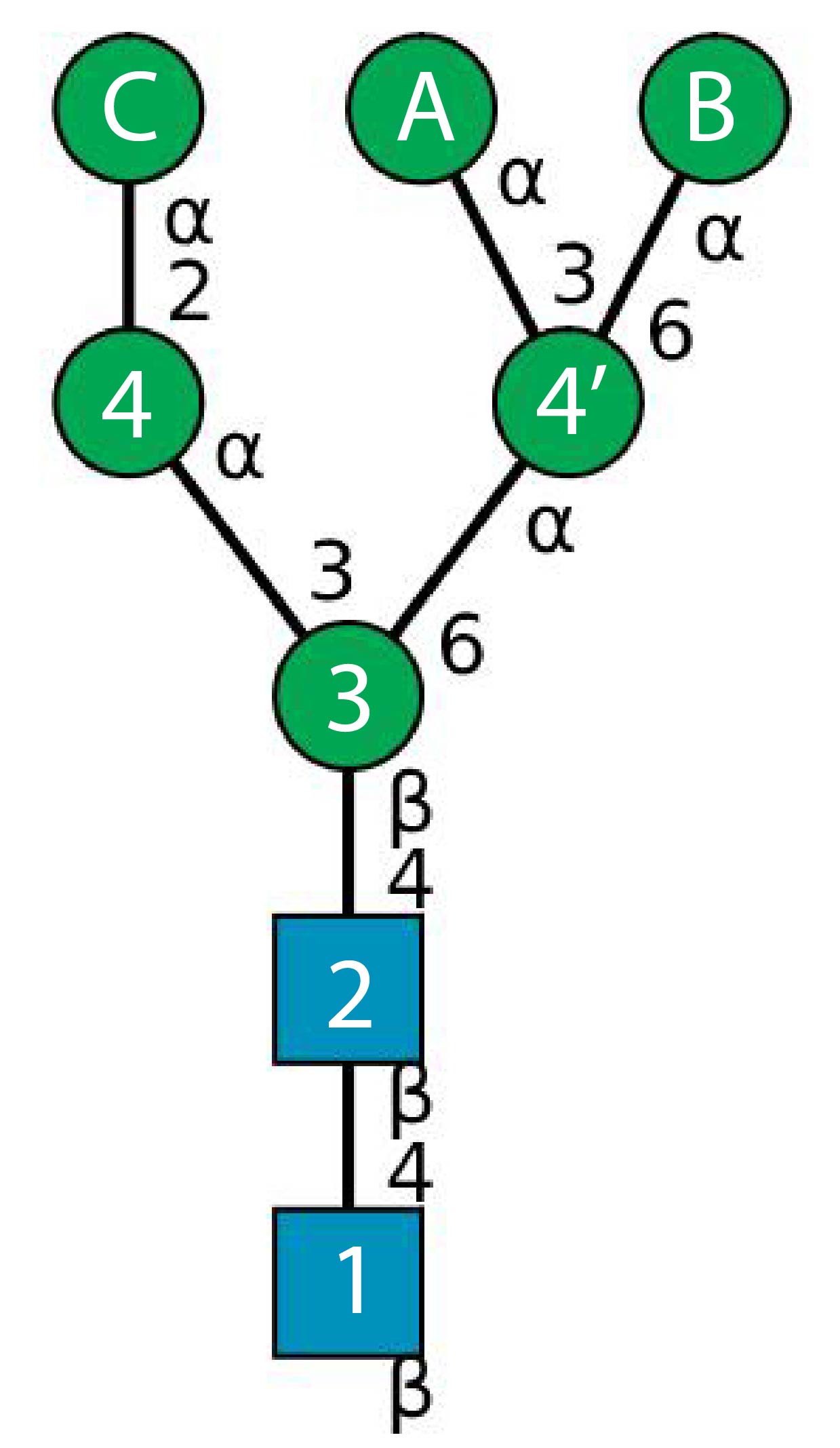_ | 7QUO | Manα1,2Man (C) | -328.2±0.8 | -143.8±0.4 | -472.0±0.9 | 231.3±0.7 | -21.7±0.4 | 209.6±0.7 | -262.5±0.7 |
|  | 7QUO | Manα1,3Man(A) | -365.2±1.0 | -139.0±0.4 | -504.2±1.1 | 259.0±0.9 | -23.1±0.0 | 236.0±0.9 | -268.3±0.8 |
|  | 7QUO | Manα1,6Man (B) | -328.0±0.9 | -103.4±0.4 | 431.4±1.0 | 187.8±0.8 | -17.5±0.0 | 170.3±0.8 | -261.0±0.8 |

# Protein-carbohydrate interactions in the crystal structures


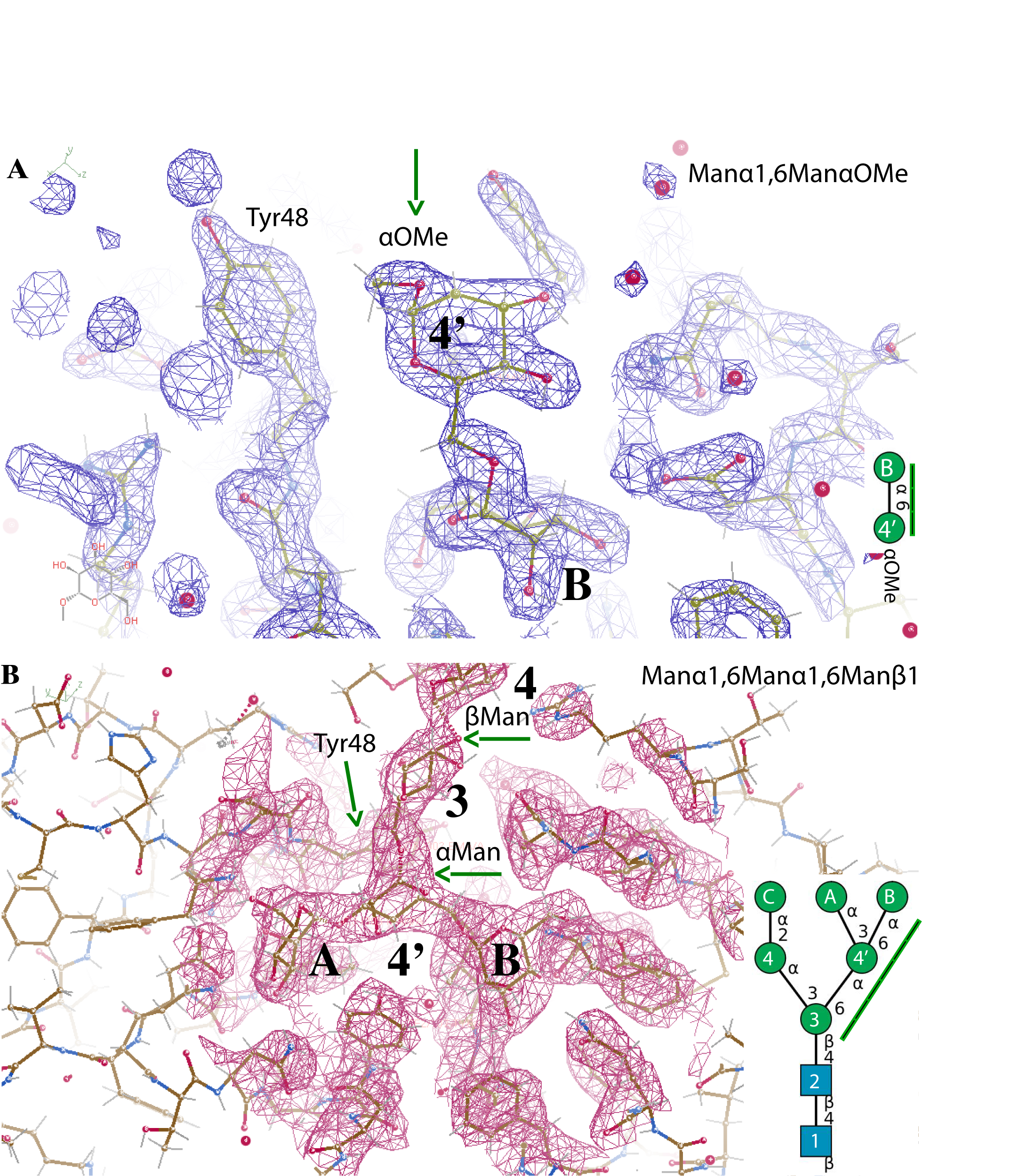


**Figure S8**. Why Manα1,6Manβ1 on the common trimannose core Man3Gn2 binds so badly. **A**. Manα1,6Manα1ΟMe bound to the FimH lectin domain (PDB entry 6GTY (9)). The reducing mannose 4’ in Manα1,6Man α1OMe has an α-anomeric configuration, a β-anomeric configuration of mannose 4’ would clash with the side chain of Tyr48. **B**. The α-anomeric configuration of mannose 4’, central in the second trimannose core of Man6Gn2 (PDB entry 7QUO), permits the bivalent binding of mannose A and mannose B of Man6Gn2 driven by the substitution of mannose 4 by mannose C.

| ***BINDING POCKET*** | 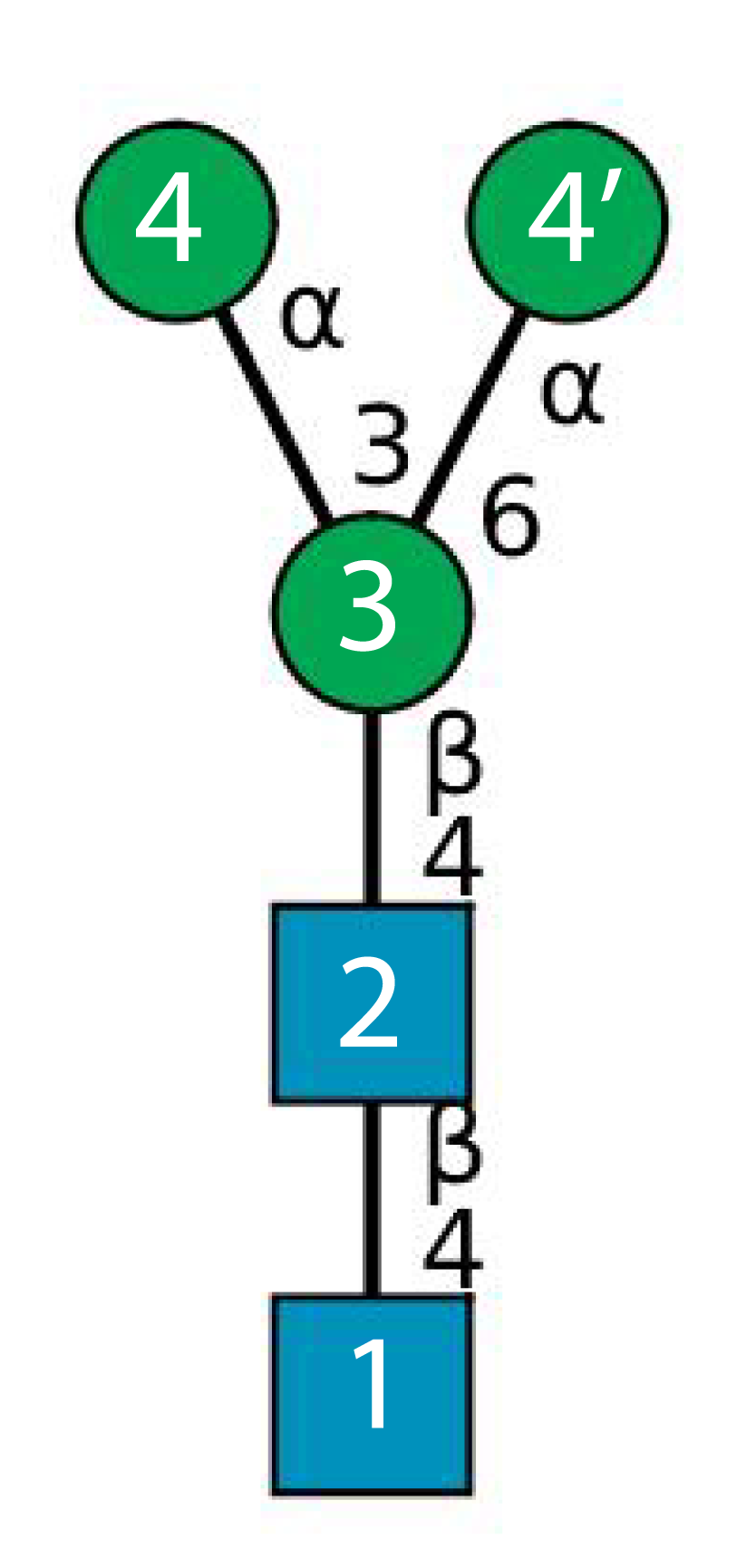 | 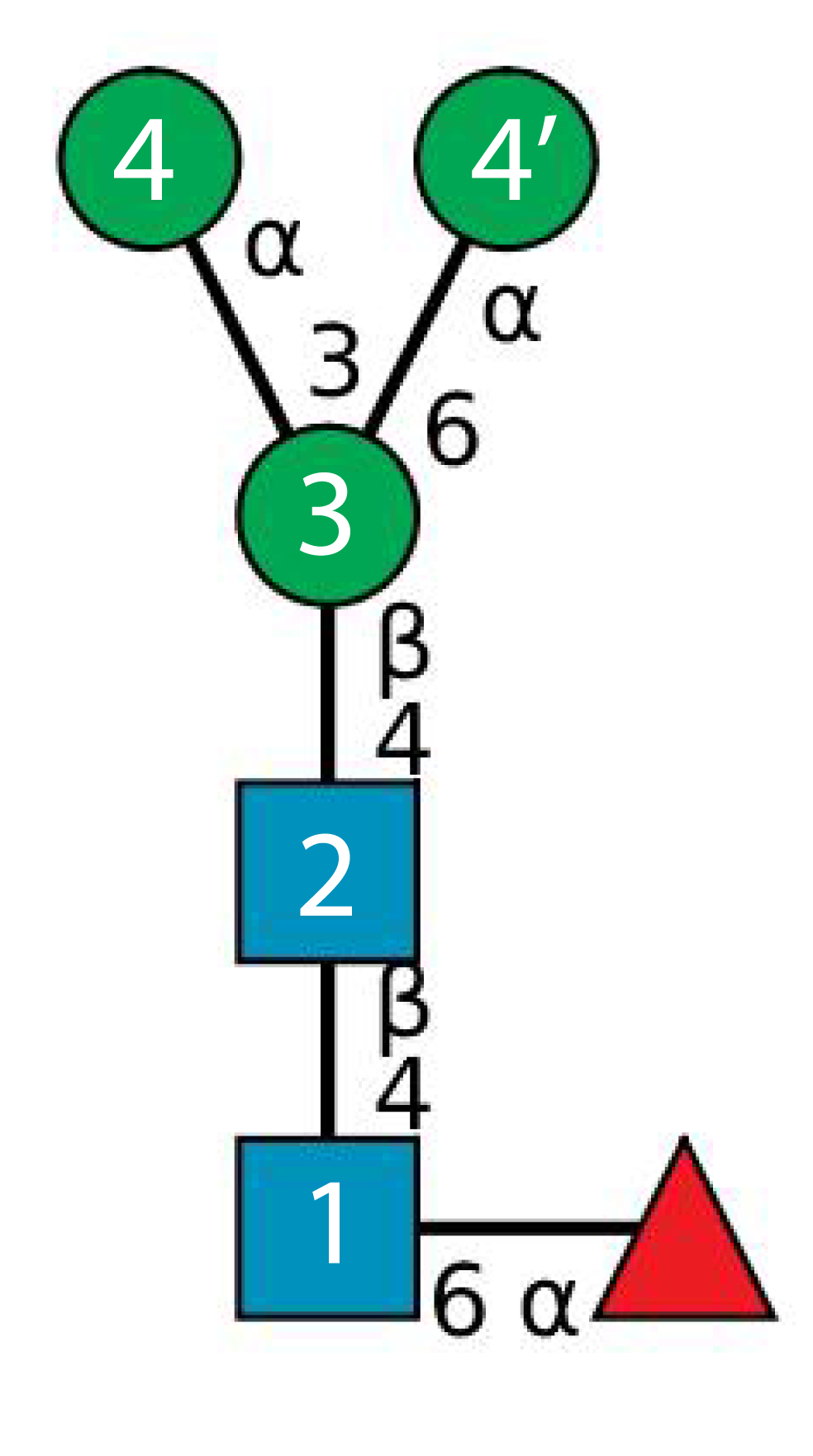 | _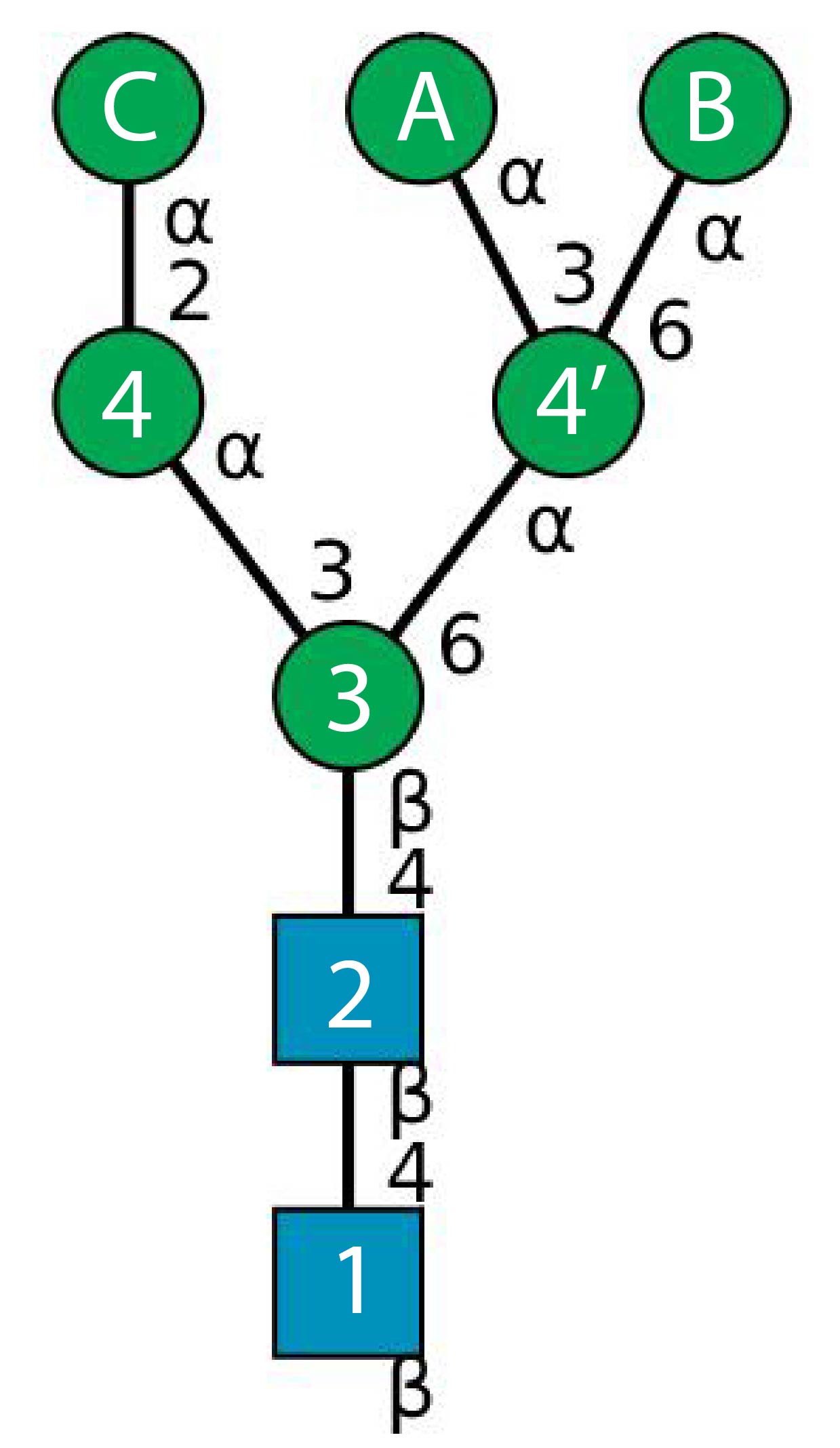_ | 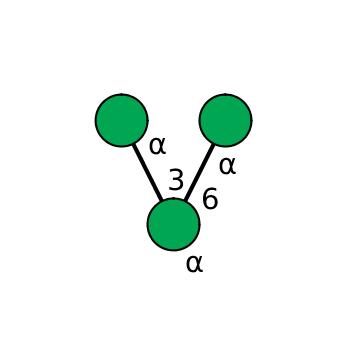 |
| --- | --- | --- | --- | --- |
|  | **2VCO** | **7BHD** | **7QUO** | **6GTV** |
| **M** | **4** | **4** | **A** | **A** |
|  | C/D_1 PHE_A/B | C/D_1 PHE_A/B | E/F_1 PHE A/B | E_1 PHE C |
|  | C/D_13 ILE A/B | C/D_13 ILE A/B | E/F_13 ILE A/B | E_ 13 ILE C |
|  | C/D_46 ASN A/B | C/D_46 ASN A/B | E/F_46 ASN A/B | E_46 ASN C |
|  | C/D_47 ASP A/B | C/D_47 ASP A/B | E/F_47 ASP A/B | E_47 ASP C |
|  | C/D_48 TYR A/B | C/D_48 TYR A/B | E_48 TYR A | E_48 TYR C |
|  | C/D_52 ILE A/B | C/D_52 ILE A/B | E/F_52 ILE A/B | E_52 ILE C |
|  | C/D_54 ASP A/B | C/D_54 ASP A/B | E/F_54 ASP A/B | E_54 ASP C |
|  | C/D_133 GLN A/B | C/D_133 GLN A/B | E/F_133 GLN A/B | E_133 GLN C |
|  | C/D_135 ASN A/B | C/D_135 ASN A/B | E/F_135 ASN A/B | E_135 ASN C |
|  | - | - | E/F_137 TYR A/B | E_137 TYR C |
|  | C/D_140 ASP A/B | C/D_140 ASP A/B | E/F_140 ASP A/B | E_140 ASP C |
|  | C/D_142 PHE A/B | C/D_142 PHE A/B | E/F_142 PHE A/B | E_142 PHE C |
| **M'** |  |  | **B** | **B** |
|  |  |  | E/F_1 PHE C/D | E_1 PHE A |
|  |  |  | E/F_13 ILE C/D | E_13 ILE A |
|  |  |  | E/F_ 46 ASN C/D | E_ 46 ASN A |
|  |  |  | E/F_47 ASP C/D | E_47 ASP A |
|  |  |  | E_ 48 TYR C | E_ 48 TYR A |
|  |  |  | E/F_52 ILE C/D | E_52 ILE A |
|  |  |  | E/F_54 ASP C/D | E_54 ASP A |
|  |  |  | E/F_ 133 GLN C/D | E_ 133 GLN A |
|  |  |  | E/F_135 ASN C/D | E_135 ASN A |
|  |  |  | E/F_140 ASP C/D | E_140 ASP A |
|  |  |  | E/F_142 PHE C/D | E_142 PHE A |
| **M+1** | **3** | **3** | **4’** | **4’** |
|  | C_13 ILE A | C/D_13 ILE A/B | E_13 ILE C | - |
|  | C/D_48 TYR A/B | C/D_48 TYR A/B | - | E_48 TYR C |
|  | - | - | - | E_48 TYR A |
|  | - | C/D_52 ILE A/B | E/F_ 52 ILE A/B | E_52 ILE A |
|  | D_52 ILE B | - | F_52 ILE D | E_52 ILE C |
|  | D_137 TYR B | - | E/F_137 TYR A/B | E_137 TYR A |
|  | - | - | - | E_137 TYR C |

**A**

| ***BINDING POCKET*** | 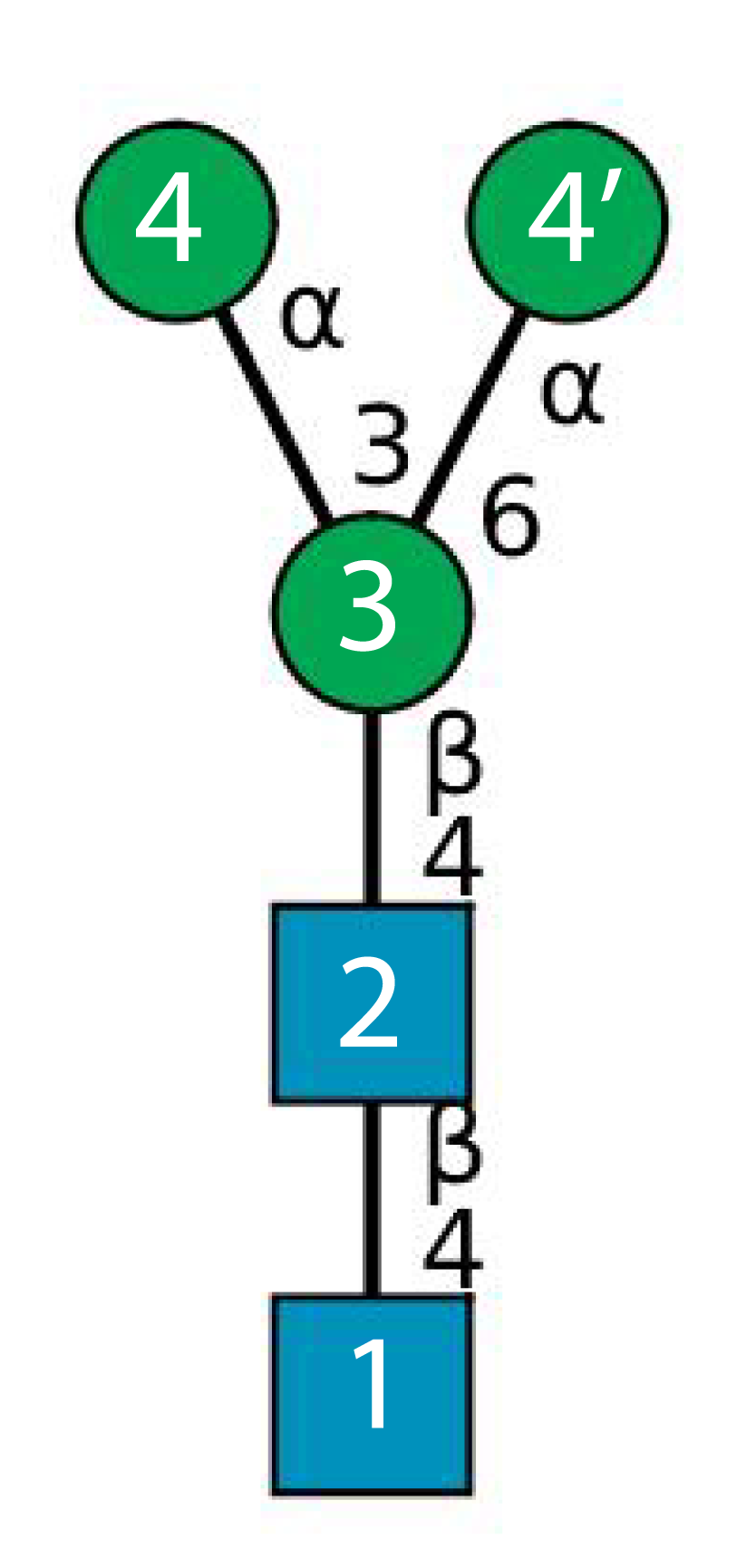 | 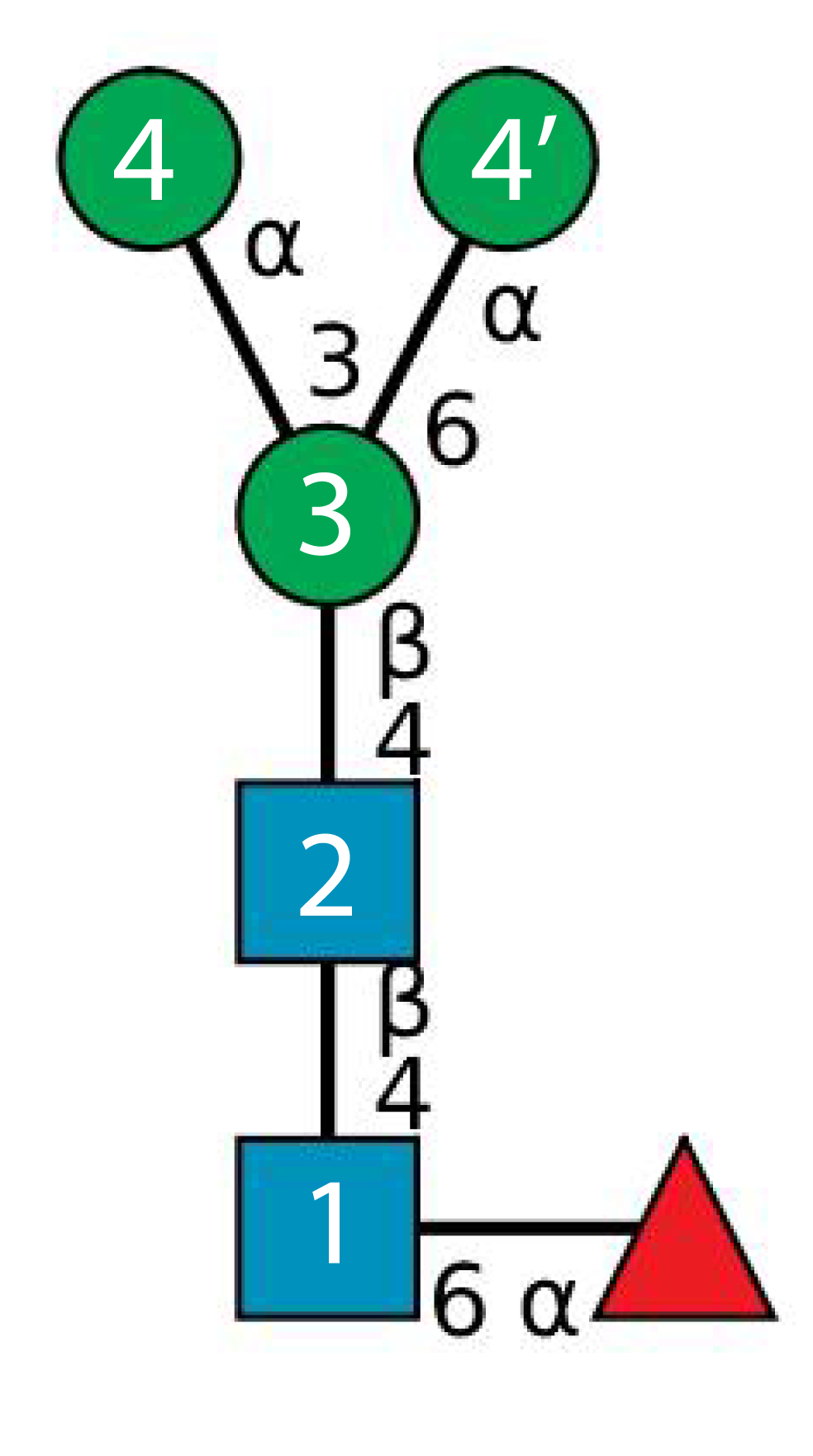 | _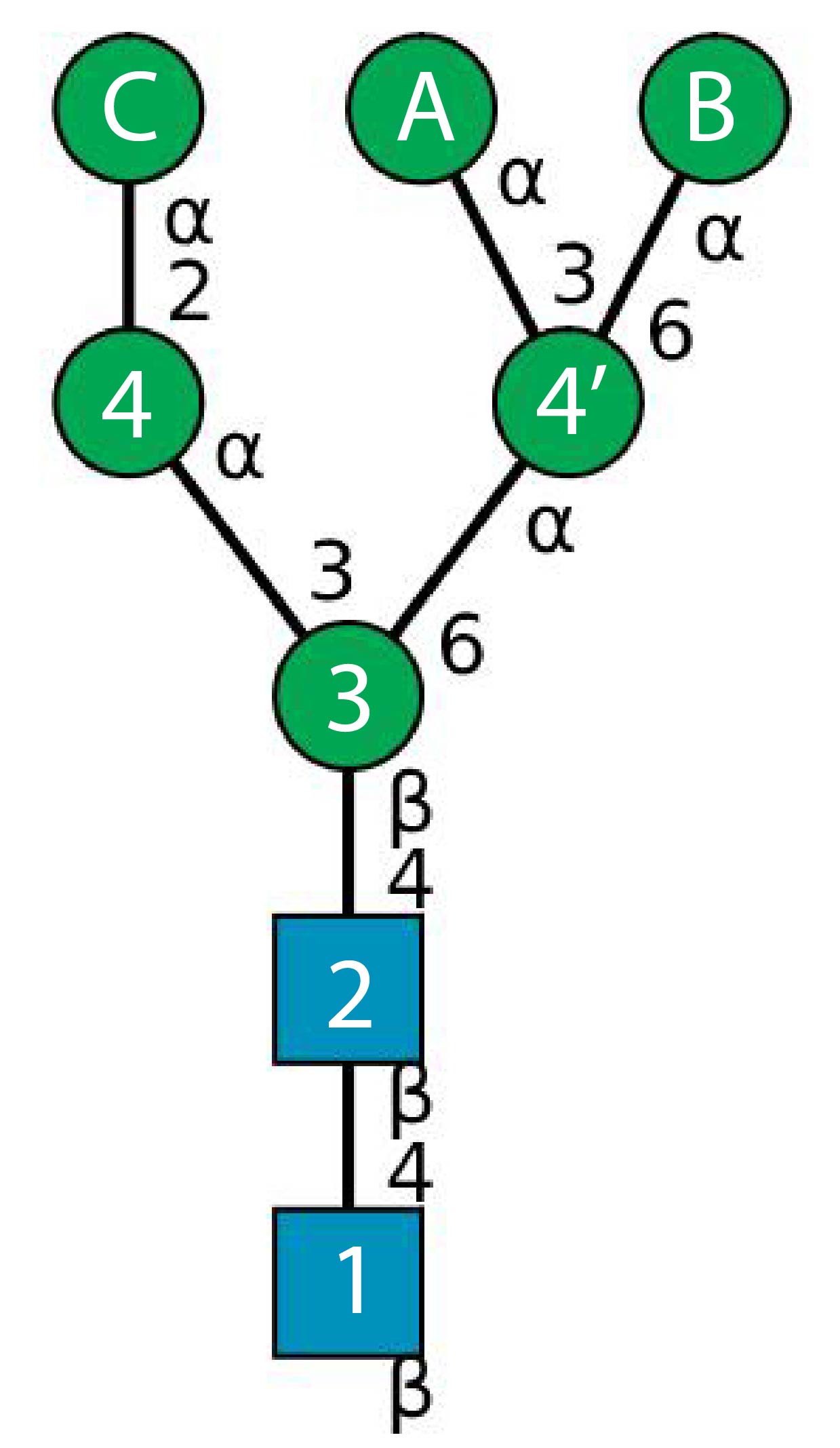_ |
| --- | --- | --- | --- |
|  | **2VCO** | **7BHD** | **7QUO** |
| **M+2** | **2** | **2** | **3** |
|  | - | - | E/F_47 ASP C/D |
|  | C/D_48 TYR A/B | C_48 TYR A | E/F_48 TYR A/B |
|  | - | - | F_48 TYR D |
|  | C/D_51 THR A/B | C/D_51 THR A/B | - |
|  | - | C/D_52 ILE A/B | - |
|  | - | - | F_98 ARG D |
|  | C/D_137 TYR A/B | C/D_137 TYR A/B | - |
|  |  |  |  |
| **M+3** | **1** | **1** | **2** |
|  | - | - | E/F_13 ILE A/B |
|  | - | - | E/F_48 TYR C/D |
|  | C/D_51 THR A/B | - | - |
|  | - | - | F_52 ILE D |
|  | - | C/D_137 TYR A/B | E/F_137 TYR C/D |
|  |  |  |  |
| **M+3'** |  |  | **4** |
|  |  |  | E/F_98 ARG C/D |
|  |  |  |  |
| **M+4** |  | **Fuc** | **1** |
|  |  | - | E/F_12 PRO A/B |
|  |  | - | E/F_13 ILE A/B |
|  |  | AC_51 THR A | - |
|  |  | BC/D_137 TYR A/B | E/F_137 TYR C/D |
|  |  |  |  |
| **M+4'** |  |  | **C** |
|  |  |  | E_98 ARG A |

**Table S4.** Interactions between the glycan and FimH compared between 4 crystal structures shown as 7BHD, 7QUO, 6GTV and 2VCO. Red coloured residues indicate hydrogen bonds, while black coloured residues indicate bumps within a distance limit < 4.0 A. M represents the monosaccharide binding site, M’ is the for the monosaccharide binding site of the second bound FimH molecules through a bivalent *N*-glycan. Subsequent monosaccharide residues are marked with +#, and with ‘ at the branch points. The chain is named as follow: C/D_13 ILE A/B where the C chain of the glycan is interacting with Ile13 from FimH chain A, and in a similar fashion the D chain of the glycan is interacting with Ile13 from FimH chain B.

**B**

**A**. M, M’ and M+1 subsites of FimH. **B**. M+2, M+3, M+3’, M+4, M+4’ subsites of FimH.

# MALDI-TOF spectrum of synthesized Man3Gn2F1[6]

**A**

**B**

**Figure S9**. **A.** MALDI-TOF spectrum of synthesized Man3Gn2F1[6] (10). **B**. Extended MALDI-TOF spectrum after purification of Man3Gn2F1[6]. The ratio of the areas of both peaks calculates to 3% of non-fucosylated glycan, which is an estimate because it is not known whether both molecules ionize the same way.

# ^1^H-NMR spectrum in D_2_O spectrum of synthesized Man3Gn2F1[6]

^
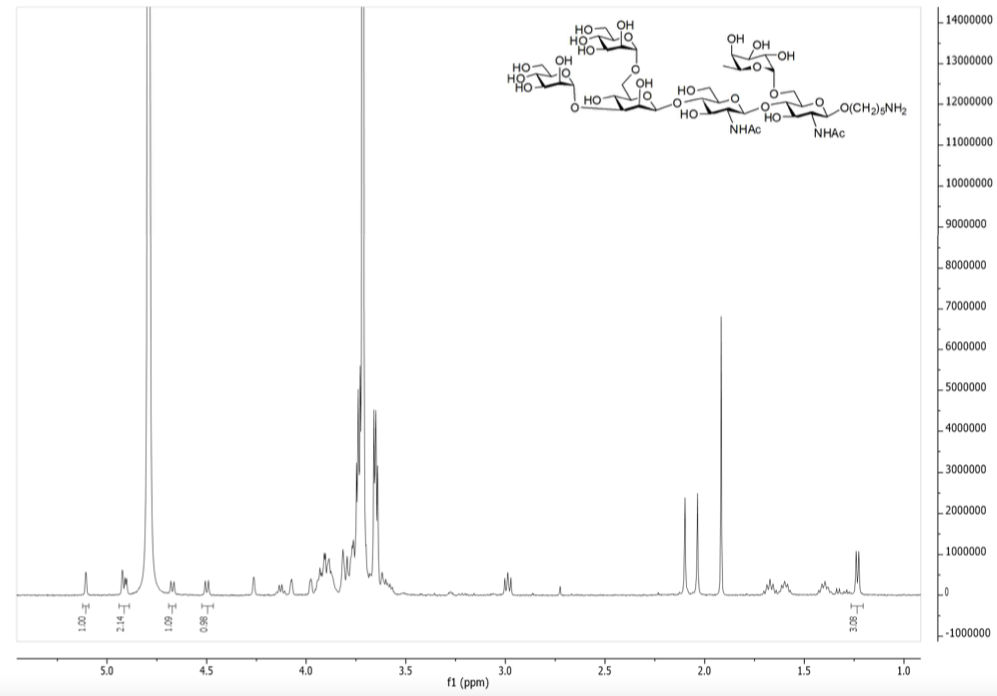
^

**Figure S10**. The ^1^H-NMR spectrum contains no traces of non-fucosylated glycan Man3Gn2, confirming its purity.

# References

1. Neelamegham, S., Aoki-Kinoshita, K., Bolton, E., Frank, M., Lisacek, F., Lutteke, T., O'Boyle, N., Packer, N. H., Stanley, P., Toukach, P., Varki, A., Woods, R. J., and SNFG-Discussion-Group. (2019) Updates to the symbol nomenclature for glycans guidelines. *Glycobiology* **29**, 620-624

2. Cheng, K., Zhou, Y., and Neelamegham, S. (2017) DrawGlycan-SNFG: a robust tool to render glycans and glycopeptides with fragmentation information. *Glycobiology* **27**, 200-205

3. Sehnal, D., Bittrich, S., Deshpande, M., Svobodova, R., Berka, K., Bazgier, V., Velankar, S., Burley, S. K., Koca, J., and Rose, A. S. (2021) Mol* Viewer: modern web app for 3D visualization and analysis of large biomolecular structures. *Nucleic Acids Res* **49**, W431-W437

4. Wellens, A., Garofalo, C., Nguyen, H., Van Gerven, N., Slattegard, R., Hernalsteens, J. P., Wyns, L., Oscarson, S., De Greve, H., Hultgren, S., and Bouckaert, J. (2008) Intervening with urinary tract infections using anti-adhesives based on the crystal structure of the FimH-oligomannose-3 complex. *PLoS ONE* **3**, e2040

5. McNicholas, S., and Agirre, J. (2017) Glycoblocks: a schematic three-dimensional representation for glycans and their interactions. *Acta Crystallogr D Struct Biol* **73**, 187-194

6. McNicholas, S., Potterton, E., Wilson, K. S., and Noble, M. E. (2011) Presenting your structures: the CCP4mg molecular-graphics software. *Acta Crystallogr D Biol Crystallogr* **67**, 386-394

7. Atanasova, M., Nicholls, R. A., Joosten, R. P., and Agirre, J. (2022) Updated restraint dictionaries for carbohydrates in the pyranose form. *Acta Crystallogr D Struct Biol* **78**, 455-465

8. Kumari, R., Kumar, R., Open Source Drug Discovery, C., and Lynn, A. (2014) g_mmpbsa--a GROMACS tool for high-throughput MM-PBSA calculations. *J Chem Inf Model* **54**, 1951-1962

9. Sauer, M. M., Jakob, R. P., Luber, T., Canonica, F., Navarra, G., Ernst, B., Unverzagt, C., Maier, T., and Glockshuber, R. (2019) Binding of the bacterial adhesin FimH to its natural, multivalent high-mannose type glycan targets. *J Am Chem Soc* **141**, 936-944

10. Brzezicka, K., Echeverria, B., Serna, S., van Diepen, A., Hokke, C. H., and Reichardt, N. C. (2015) Synthesis and microarray-assisted binding studies of core xylose and fucose containing *N*-glycans. *ACS Chem Biol* **10**, 1290-1302
